# Alpha radionuclide-chelated radioimmunotherapy promoters enable local radiotherapy/chemodynamic therapy to discourage cancer progression

**DOI:** 10.1186/s40824-022-00290-6

**Published:** 2022-09-08

**Authors:** Jiajia Zhang, Feize Li, Yuzhen Yin, Ning Liu, Mengqin Zhu, Han Zhang, Weihao Liu, Mengdie Yang, Shanshan Qin, Xin Fan, Yuanyou Yang, Kun Zhang, Fei Yu

**Affiliations:** 1 Department of Nuclear Medicine, Shanghai Tenth People’s Hospital, Tongji University School of Medicine, No. 301 Yan-chang-zhong Road, Shanghai, 200072 People’s Republic of China; 2 Institute of Nuclear Medicine, Tongji University School of Medicine, No. 301 Yan-chang-zhong Road, Shanghai, 200072 People’s Republic of China; 3 Department of Medical Ultrasound and Central Laboratory, Ultrasound Research and Education Institute, Shanghai Tenth People’s Hospital, Tongji University School of Medicine, No. 301 Yan-chang-zhong Road, Shanghai, 200072 People’s Republic of China; 4 Key Laboratory of Radiation Physics and Technology of the Ministry of Education, Institute of Nuclear Science and Technology, Sichuan University, Chengdu, 610064 People’s Republic of China

**Keywords:** Targeted alpha therapy, Immune activation, Chemodynamic therapy, Immune checkpoint blockade, Tumor relapse inhibition

## Abstract

**Background:**

Astatine-211 is an α-emitter with high-energy α-ray and high cytotoxicity for cancer cells. However, the targeted alpha therapy (TAT) also suffers from insufficient systematic immune activation, resulting in tumor metastasis and relapse. Combined immune checkpoint blockade (ICB) with chemodynamic therapy (CDT) could boost antitumor immunity, which may magnify the immune responses of TAT. This study aims to discourage tumor metastasis and relapse by tri-model TAT-CDT-ICB strategy.

**Methods:**

We successfully designed Mn-based radioimmunotherapy promoters (^211^At-ATE-MnO_2_-BSA), which are consisting of ^211^At, MnO_2_ and bovine serum albumin (BSA). The efficacy of ^211^At-ATE-MnO_2_-BSA was studied as monotherapy or in combination with anti-PD-L1 in both metastatic and relapse models. The immune effects of radioimmunotherapy promoters on cytotoxic T lymphocytes and dendritic cells (DCs) were analyzed by flow cytometry. Enzyme-linked immunosorbent assay and immunofluorescence were used to explore the underlying mechanism.

**Results:**

Such radioimmunotherapy promoters could not only enhance the therapeutic outcomes of TAT and CDT, but also induce robust anti-cancer immune activity by activating dendritic cells. More intriguingly, ^211^At-ATE-MnO_2_-BSA could effectively suppress the growths of primary tumors and distant tumors when combined with immune checkpoint inhibitors.

**Conclusions:**

The tri-model TAT-CDT-ICB strategy provides a long-term immunological memory, which can protect against tumor rechallenge after eliminating original tumors. Therefore, this work presents a novel approach for TAT-CDT-ICB tri-modal cancer therapy with repressed metastasis and relapse in clinics.

**Supplementary Information:**

The online version contains supplementary material available at 10.1186/s40824-022-00290-6.

## Introduction

Radiotherapy (RT) includes internal radiotherapy (IRT) and external-beam radiotherapy (EBRT), both of which highlight ionizing radiation beams such as X-ray, β-ray, or α-ray directly kill cancer cells and boost antitumor immunity [1–3]. IRT has attracted increasing interest since it features high safety and efficacy, and has been widely used to activate antitumor immunity [3]. However, the current nuclides predominantly focused on β-particles, where β-particles inevitably pose physiological toxicity and radiation resistance due to the presence of a hypoxic tumor microenvironment [4, 5]. Compared to β-particles, α-particles are much preferable because they have high linear energy transfer (> 50 keV/μm), which determined that they can lead to more DNA double-strand injures and relative biological efficiency within a much lower radiation dose. More importantly, the alpha nuclide-based IRT is independent on cellular oxygenation [6, 7]. Unfortunately, medically-useful α-emitters remain inaccessible and the activated immune responses are insufficient, hampering their clinical expansion.

Chemodynamic therapy (CDT) is a promising therapy by utilizing Fenton or Fenton-like reactions to modulate the immunogenic tumor microenvironment [8]. Previous study showed that Fe-based CDT could induce immunogenic cell death and promote the release of tumor-associated antigens (TAAs), furtherly boosting the systemic immune responses [9] . However, single CDT strategy alone also is not sufficient to activate immune responses for suppressing tumor metastasis and recurrence, which usually needs combined therapy such as immunotherapy, radiotherapy, etc. Tumor metastasis and recurrence caused 90% of patient deaths in clinic practices [10, 11], and immunotherapy was identified as the most promising mean to eradicate tumors and repress tumor metastasis. Immunotherapy can stimulate immune responses to assault distant tumors and even induce robust immune memory effects [4]. However, the inherently immunosuppressive tumor microenvironment (ITM), immune escape, and immune desert render malignancy into a cold one featuring low mutation burdens, low neoantigen burden and inadequate infiltrated effector T cells [12]. Typically, although immune checkpoint blocking (ICB) therapy has made significant progress in clinics [13–15], only less than 20% of cancer patients could benefit from ICB due to the ITM [16]. Regarding this, immunotherapy synergy with other therapeutic approaches was highlighted to activate systematic immune responses and mitigate ITM [17, 18]. Inspiringly, the marriage of alpha radionuclide-based IRT with ICB is also expected to arouse potent immune responses and repress tumor metastasis.

In this report, ^211^At-tethered radioimmunotherapy promoters were established to simultaneously achieve IRT and chemodynamic therapy, wherein bovine serum albumin (BSA)-coated MnO_2_ (MnO_2_-BSA) nanoparticles were firstly yielded via a well-established protein biomimetic mineralization method [19]. ^211^At as the most suitable candidate for targeted alpha therapy (TAT) could be conjugated to MnO_2_-BSA nanoparticles via condensation reaction by the bifunctional linker, i.e., N-succinimidyl 3-trimethylstannyl-benzoate (ATE). The short half-life of ^211^At determines that ^211^At will not cause medical problems that other α-emitting radionuclides such as ^223^Ra and ^225^Ac suffer from [20, 21]. Besides serving as carriers, MnO_2_-BSA enables chemodynamic therapy process to generate •OH, which has been documented to directly kill tumor cells and simultaneously enhance ICB.

**Scheme 1:**
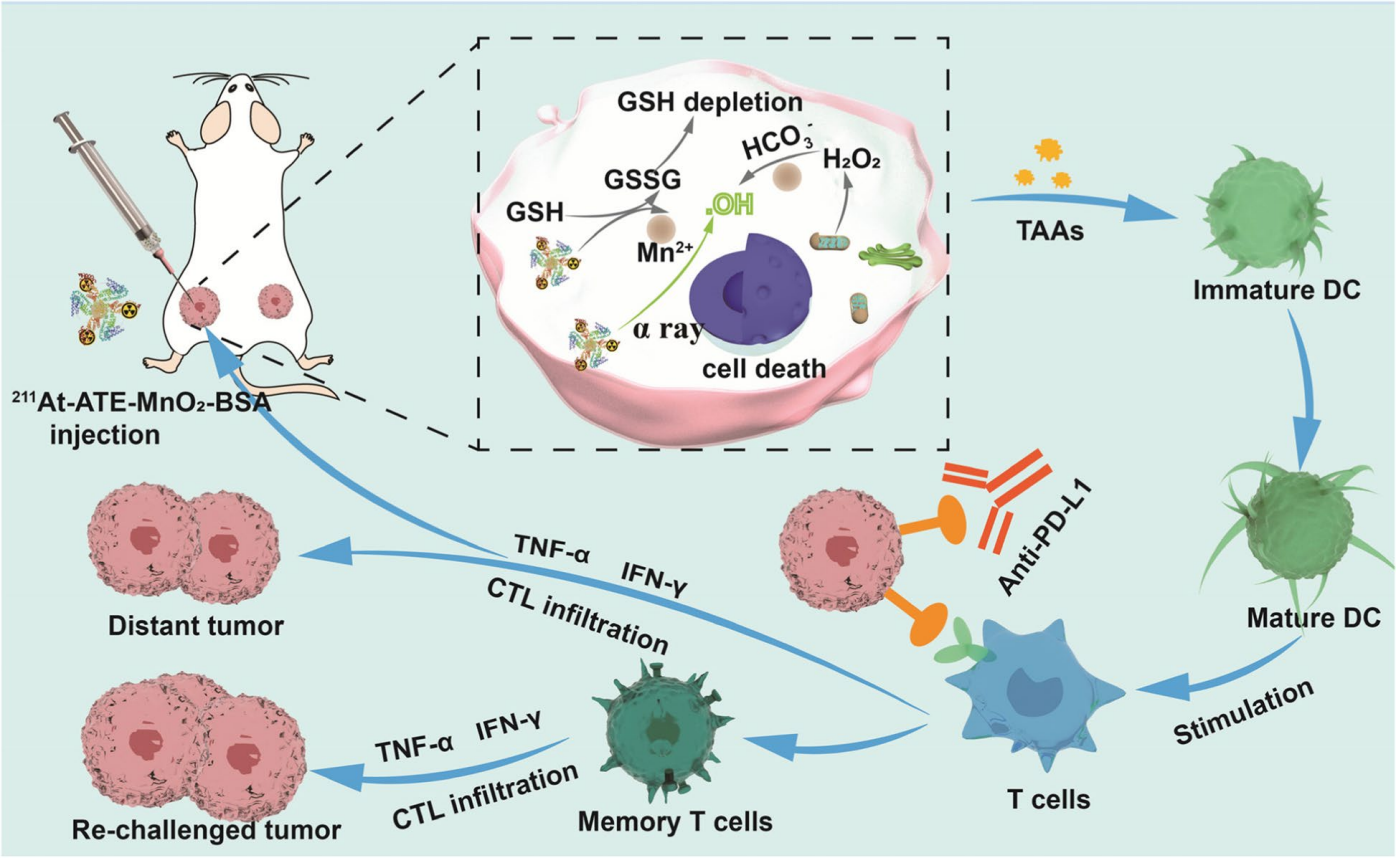
The schematic diagram and action mechanism of radioimmunotherapy promoters-augmented synergistic TAT/CDT/ICB therapy

It has been documented that reactive oxygen species (ROS) as well as their killing effects could trigger more tumor-associated antigens release, modulate ITM and activate systematic immune responses [9, 22–24]. In light of it, the combined therapy consisting of CDT and TAT in ^211^At-ATE-MnO_2_-BSA could not only extraordinarily ameliorate the tumor progression through TAT and CDT both in vitro and vivo, but also could elicit the robust antitumor immune responses and increase the infiltration of cytotoxic T lymphocytes (CTLs), further repressing tumor cells. Especially after integrating with anti-PD-L1-enabled ICB**,** the tri-modal TAT/CDT/ICB combined therapy in ^211^At-ATE-MnO_2_-BSA radioimmunotherapy promoters effectively ablated the primary tumor, and more importantly gave rise to long-term immunological memory effects to inhibit distant metastasized ones. Taken all together, this study explored a novel strategy to establish a TME-activated therapy based on ^211^At-ATE-MnO_2_-BSA, which achieved synergistic TAT/CDT/ICB with remarkable efficiency **(**Scheme 1**)**.

## Methods

### Materials

The radioisotope ^211^At was produced via ^209^Bi (α, 2n) ^211^At reaction through CS-30 cyclotron according to the published protocol [25]. Bovine serum albumin (BSA), Manganese chloride tetrahydrate (MnCl_2_·4H_2_O), and sodium hydroxide (NaOH) were from Sigma-Aldrich. Roswell park memorial institute (RPMI) 1640 medium, penicillin-streptomycin, and fetal bovine serum (FBS) were obtained from Gbico. The cell counting kit-8 (CCK-8) was obtained from Biosharp. The anti-mouse PD-L1 antibody was obtained from BioXcell (clone:10F.9G2). APC anti-mouse CD45(Catalog: 103111), FITC anti-mouse CD3 (Catalog: 100203), BV421™ anti mouse CD4(Catalog: 100437), APC/Cy7 anti-mouse CD8a (Catalog: 100714), PE anti-mouse FOXP3 (Catalog: 126403), FITC anti-mouse CD11c (Catalog: 117305), PE anti-mouse CD86 (Catalog:105007), APC anti-mouse CD80 (Catalog: 104713), PE/Cy7 anti-mouse CD45 (Catalog: 103113), PE anti-mouse CD44(Catalog:103023) and BV421- anti-mouse CD62L (Catalog: 104435) antibodies were obtained from Biolegend, Tumor necrosis factor alpha (TNF-α) and interferon gamma (IFN-γ) ELISA kit was purchased from Multisciences Biotech, Co., Ltd.

### Synthesis of MnO_2_-BSA nanoparticles

The MnO_2_-BSA were prepared following a BSA-constrained biomineralization strategy. Briefly, a BSA solution (125 mg, 50 mL) and an aqueous MnCl_2_·4H_2_O solution (0.1 M, 250 μL) were mixed and stirred at 37 °C for 3 min. Subsequently, 500 μL NaOH solution (1 M) was introduced to adjust the pH value to 11 ~ 12. After 6 h of stirring at 37 °C, the brown mixture was collected and dialyzed (MWCO = 8000 ~ 14,000 Da) with deionized water for 24 h to remove redundant Mn^2+^. Finally, the powder was collected after lyophilization and stored at 4 °C for further use.

### Characterization of MnO_2_-BSA

Transmission electron microscope (Tecnai G2 F20 S-TWIN, Netherlands) was used to observe the morphology and size of the MnO_2_-BSA. The size distribution of MnO_2_-BSA was monitored by dynamic light scattering (DLS) using a Malvern Zetasizer Nano ZS instrument. The UV-vis absorption spectrum of MnO_2_-BSA were measured by an UV-vis spectrophotometer (UV-2450, Shimadzu, Japan). Elemental mappings and energy dispersive X-ray spectroscopy (EDS) were acquired from FEI Talos F200X. X-ray photoelectron spectroscopy (XPS) was used to analyze the valence state of Mn in MnO_2_-BSA by Thermo Kalpha. The Mn content were quantified on the inductively coupled plasma-optical emission spectrometry (ICP-OES, Agilent 730).

### The generation of ·OH with MnO_2_-BSA NPs

To measure the generation of ·OH, the MnO_2_-BSA NPs were mixed with GSH solution (10 mM) at different pH (7.4, 6.5 and 5.0). Subsequently, 10 μg/mL methylene blue (MB), different concentration H_2_O_2_ (1 mM，4 mM, 8 mM) were mixed. After different time incubation, the ·OH generation ability was evaluated by MB degradation via the change in absorbance. In addition, an electron paramagnetic resonance (EPR) spectrometer was also used to detect the existence of ·OH.

### Radioisotope labeling and labeling stability assay

N-succinimidyl 3-trimethylstannyl benzoate (ATE) (dissolved in DMSO) and MnO_2_-BSA (dissolved in PH = 9 Na_2_CO_3_ buffer) were mixed and incubated at room temperature for 30 min. Subsequently, the product was purified using an Amicon filters (MWCO = 30 kDa) to remove excess ATE. Subsequently, ATE-MnO_2_-BSA, N-iodosuccinimide (NIS, 3 mg/mL in MeOH) and ^211^At (500 μCi) solution was mixed and allowed to stand for 30 min at room temperature. The reacted solution was purified using an Amicon filters (MWCO = 30 kDa) to remove redundant ^211^At till no detachable radioactivity in the filtrate solution [26]. The in vitro stability of ^211^At-ATE-MnO_2_-BSA was determined with paper chromatography through Whatman No.1 filter paper. Briefly, ^211^At-ATE-MnO_2_-BSA (10 μL) was added to 100 μL of PBS (PH = 7.4) or 10% FBS and incubated at 37 °C for 0 h, 5 h, and 24 h. At each time point, 1 μL of mixture was placed Whatman No.1 filter paper and was allowed to be evaporated spontaneously. Then, we put it into the developing tank with developing solvent to separate ^211^At-ATE-MnO_2_-BSA from free ^211^At. When the chromatography liquid reaches the front, the filter paper was taken out and dried at room temperature. Finally, we cut the paper into 1 cm sections, and put the paper pieces into the test tube in turn. The radioactivity count of each paper was measured in the counter.

### Cell cytotoxicity assay

The breast cancer 4 T1 cells and colorectal cancer CT26 cells were originally obtained from the Shanghai Institute of Cells, Chinese Academy of Sciences, and cultured under recommended conditions. 4 T1 cells and CT26 cells were pre-seeded in a 96-well plate with a density of 5 × 10^3^ cells per well overnight to allow the attachment of cells. Free ^211^At and ^211^At-ATE-MnO_2_-BSA were co-cultured with 4 T1 cells and CT26 cells at different radioactivity for 12 h. Subsequently, the cell viability was measured according to the CCK8 assay kit protocol at the wavelength of 450 nm.

### Immunofluorescence staining of γ-H2AX

Breast cancer 4 T1 cells were seeded in 6-well plates with densities of 3 × 10^4^ cells per dish. After 24 h, cells were treated with MnO_2_-BSA, free ^211^At, and ^211^At-ATE-MnO_2_-BSA for 12 h. Then, the cells were dealt with γ-H2AX and DAPI and imaged by a fluorescence microscope (Olympus).

### Comet assay

The 4 T1 cells were seeded into 6-well plates. After the cells were adhered onto wall, different treatments were conducted as follows, e.g., (1) control, (2) MnO_2_-BSA, (3) ^211^At, (4)^211^At-ATE-MnO_2_-BSA. After 12 h, the cells of each group were collected by centrifugation and washed with PBS to obtain the cell suspension. 10 μL of cell suspension was taken out and mixed with 100 μL of comet agarose. The mixture was dripped onto the 6-well comet slide. Subsequently, lysis, unrotation, and electrophoresis were performed in turn. After electrophoresis, the cells were stained with propidium iodide for 10 min and washed twice. Finally, the degree of DNA damage in cells was photographed by fluorescence microscopy.

### Tumor models and efficacy evaluation

All animal experiments were approved by Animal Welfare Ethics Committee of Shanghai Tenth People’s Hospital with an approval number (ID: SHDSYY-2021-3429-0746). Female BALB/c mice aged 6–8 weeks were purchased from Chengdu Dossy Experimental Animals Co., Ltd. To construct the tumor-bearing mice model, 1 × 10^6^ 4 T1 cells and CT26 cells were subcutaneously injected into the back of BALB/c mice. Mice were administrated with potassium iodide solution (1%) for 7 days to saturate the thyroid and decrease the uptake of the thyroid. To investigate bio-distribution of ^211^At-ATE-MnO_2_-BSA, 4 T1 tumor-bearing mice and CT26 tumor-bearing mice were intratumorally injected with ^211^At-ATE-MnO_2_-BSA at radioactive dose of 15 micro Curie (μCi). Then mice were sacrificed at 12 h, tumors and organs were collected and weighed, and the radioactivity was tested. For subcutaneous tumors inhibition, mice bearing subcutaneous 4 T1 tumors were randomly divided into four groups including control, MnO_2_-BSA (i.t., 90 μM), free ^211^At (i.t., 15 μCi), and ^211^At-ATE-MnO_2_-BSA (i.t.,15 μCi) respectively. The tumor and body weight were measured every second day. And the tumor volume was calculated using the following equation: volume = width^2^ × length/2. The 4 T1 tumors in each group were harvested on day 14 post treatment to perform the H&E staining and TUNEL staining.

For distant tumors inhibition, 4 T1 cells and CT26 cells were inoculated onto both flanks of every BALB/c mouse back. The left injected with 1 × 10^6^ cells were regarded as the primary tumor and the right injected with 5 × 10^5^ cells were set as the distant tumor. Mice were administrated with 1% KI solution for 7 days before the treatment and were divided into four groups randomly, including (1) control, (2) anti-PD-L1, (3) ^211^At-ATE-MnO_2_-BSA, (4) ^211^At-ATE-MnO_2_-BSA + anti-PD-L1. For the primary tumor, ^211^At-ATE-MnO_2_-BSA was intratumorally injected into animals at the dose of 15 μCi. Anti-PD-L1 antibodies were intraperitoneally injected with anti-PD-L1 antibodies (75 μg per mouse) at 1, 3 and 5 days. The growth of primary/distant tumors and body weight was monitored every two days. To explore the antitumor immune effect, CT26 tumors were collected and digested to conduct single cell suspensions. The cell suspensions were stained with the corresponding antibodies to identify the activated T cells (FITC-anti-mouse CD3 and APC-Cy7-anti-mouse CD8 antibodies) and Treg cells (BV421-anti-mouse CD4 and PE-anti-mouse Foxp3 antibodies), finally analyzed by flow cytometry. In addition, interferon gamma (IFN-γ, Multi sciences) and tumor necrosis factor alpha (TNF-α, Multi sciences) in the serum were analyzed by ELISA assay. To study the immune memory effect, 1 × 10^6^ CT26 cells were inoculated onto the right flanks of every BALB/c mouse. Then, the tumors were eliminated by ^211^At-ATE-MnO_2_-BSA plus anti-PD-L1, and five sex- and age-matched mice were chosen as controls. The secondary CT26 cells were inoculated in both groups after 28 days. The size of secondary tumors was assessed once per three days to monitor survival of mice. At the end of this experiment, the spleen cells of the mice were collected and identified by FCM analysis.

### Ex vivo analysis of dendritic cells

To study in vivo DCs stimulation of ^211^At-ATE-MnO_2_-BSA, tumors were obtained from CT26 tumor-bearing mice after 3 days post-treatments including control, MnO_2_-BSA (i.t., 90 μM), free ^211^At (i.t., 15 μCi), and ^211^At-ATE-MnO_2_-BSA (i.t.,15 μCi) to acquire single cell suspension. After that, the DCs were stained FITC anti-mouse CD11c (Catalog: 117305), PE-anti-mouse CD86 (Catalog:105007), APC anti-mouse CD80 (Catalog: 104713) antibodies for flow cytometry assay.

### In vivo biosafety evaluation

The blood samples were collected for liver function and kidney function analysis. At the same time, the major organ including lungs, heart, liver, spleen, and kidneys were harvested for HE staining using a standard protocol and finally observed under a microscope.

### Statistical analysis

The statistical analysis was carried out with Origin2019 and Graphpad prism8. All experimental data were expressed in this manuscript as mean ± standard deviation. The statistical significance of the differences was determined by t-test. The asterisks indicate significant differences (* means *P* < 0.05, ** means *P* < 0.01, *** means *P* < 0.001).

## Results

### Synthesis and characterization of MnO_2_-BSA NPs

**Fig. 1:**
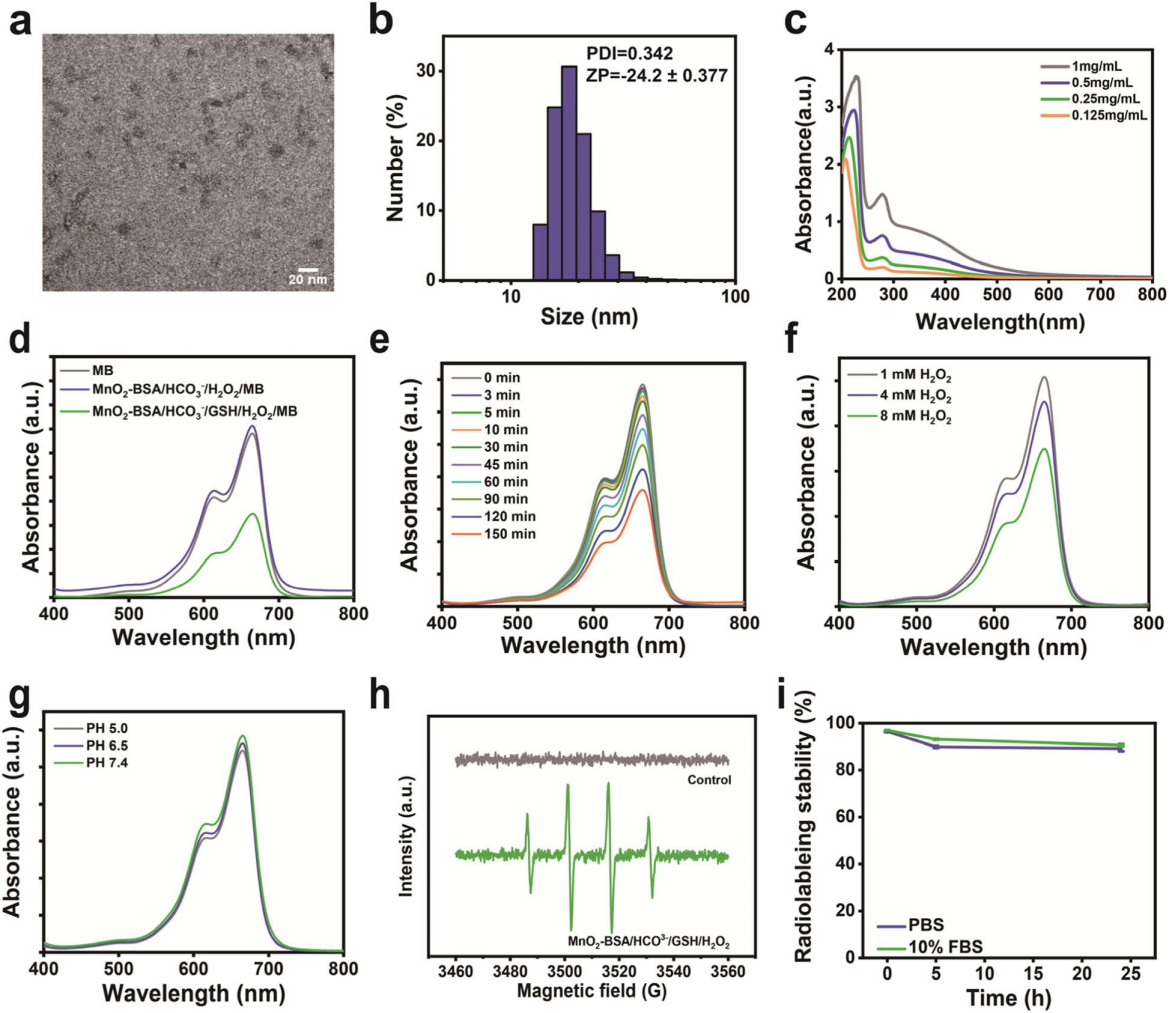
Characterization of MnO_2_-BSA NPs. **(a)** TEM images of MnO_2_-BSA. **(b)** Size distribution of MnO_2_-BSA determined by DLS in PBS. **(c)** UV–vis absorbance spectra of MnO_2_-BSA with different concentrations in PBS. **(d)** UV-Vis absorption spectra of MB after degradation by the MnO_2_-BSA -mediated Fenton-like reaction in different solutions. **(e)** The degradation process of MB at different time points. **(f)** The degradation process of MB under different concentration of H_2_O_2_. **(g)** The degradation process of MB under different PH. **(h)** EPR spectra of different groups, where the 5,5-dimethyl-1-pyrroline N-oxide (DMPO) served as the spin trapping agents to detect the •OH generation during the reaction. **(i)** Radiolabeling stability of ^211^At-ATE-MnO_2_-BSA in PBS and 10% FBS

The MnO_2_-BSA radioimmunotherapy promoters were synthesized via an environment-friendly biomimetic mineralization method, and the obtained MnO_2_-BSA nanoparticles show relatively uniform sizes and well-defined shapes with an average diameter of ~ 20 nm (Fig. 1a), which is further verified by dynamic light scattering (DLS). The hydrodynamic diameter of MnO_2_-BSA is around 20 nm (polydispersity index, PDI = 0.342) and with a negative surface charge (− 24.2 mV) (Fig. 1b). The MnO_2_-BSA nanoparticles show an evident characteristic absorbance peak within 200 nm–300 nm, indicating the successful synthesis (Fig. 1c). The energy-dispersive spectroscopy (EDS) confirms the presence of main components (Mn and O) (Fig. S1a), and the element mappings show co-existence of Mn and O elements in the structure of MnO_2_-BSA (Fig. S1b), further indicating the successful construction of MnO_2_-BSA NPs. Additionally, the X-ray photoelectron spectroscopy (XPS) shows + 4 valence of Mn atoms in MnO_2_-BSA NPs (Fig. S2), wherein the content of Mn elements is 21% in MnO_2_-BSA according to the ICP-OES. MnO_2_-BSA can react with H_2_O_2_ to produce •OH through a Fenton-like reaction with the help of HCO_3_^−^. Methylene blue (MB) was selected as an indicator to assess the •OH generation since MB could be degraded by •OH [27, 28]. Apparently, the absorbance of MB sharply declines when they are incubated with the mixture of MnO_2_-BSA, H_2_O_2_ and GSH. In contrast, H_2_O_2_ and MnO_2_-BSA in the absence of GSH fail to significantly affect MB absorbance even though they are proceeded under the help of HCO_3_^−^ (Fig. 1d), suggesting poor chemical dynamic action. As the reaction time elapses, MB degradation is increased (Fig. 1e), and the MB degradation behavior displays an H_2_O_2_ concentration-dependent manner (Fig. 1f). The reaction rates under different pH values (PH = 5.0, 6.5 and 7.4) were also detected by the MB degradation method, and results show that the reaction rates at different pH values have no obvious difference, indicating the high efficiency of MnO_2_-BSA-mediated CDT (Fig. 1g). Electron paramagnetic resonance (EPR) spectrometer that utilized 5,5-dimethyl-1-pyrroline N-oxide (DMPO) as the •OH capturing agent to confirm CDT occurrence and •OH production [18, 29]. The control group is disabled to produce •OH (Fig. 1h), while the group of MnO_2_-BSA containing H_2_O_2_ and GSH generates massive •OH, as evidenced by the emergence of characteristic peaks of •OH**.** More importantly, radiolabelling stability assay shows that ^211^At-ATE-MnO_2_-BSA radioimmunotherapy promoters are equipped with high stability in PBS and 10% FBS over 24 h (Fig. 1i).

### In vitro direct inhibition by the combined CDT and TAT

ROS has been documented to correlate with different biological activities [30–33], especially in which ROS could directly destroy tumor cells and activate immune responses [34]. As the CCK8 assay reveals, the low radioactive dose of ^211^At-ATE-MnO_2_-BSA radioimmunotherapy promoters efficiently suppresses 4 T1 cells and CT26 cells. Notably, as the radioactive dose of ^211^At-ATE-MnO_2_-BSA just reaches 0.48 μCi, the cell viabilities of 4 T1 and CT26 are dramatically decreased (Fig. S3). The higher inhibition rate of ^211^At-ATE-MnO_2_-BSA against CT26 than 4 T1 is attributed to that CT26 cells are more sensitive to radiation. It is well known that TAT can generate free radicals to cause double DNA damages. To evaluate the DNA destruction caused by TAT, γ-H2AX that positively correlates with DNA damages was assessed by immunofluorescence staining [35]. It is found that the ^211^At-ATE-MnO_2_-BSA group shows the strongest red fluorescence signal and intensity, while the free ^211^At and MnO_2_-BSA receive few or no red fluorescence (Fig. S4). Comet assay was performed to further evaluate the DNA damages in each group, where DNA damages were assessed by the tail length of fluorescent DNA staining [36]. Results show that the cells treated with ^211^At-ATE-MnO_2_-BSA receive the most evident comet tails (Fig. S5). These results suggest that ^211^At in ^211^At-ATE-MnO_2_-BSA can promote ROS production for destroying DNA and killing tumor cells.

### In vivo combined CDT and TAT inhibit tumor progression

**Fig. 2:**
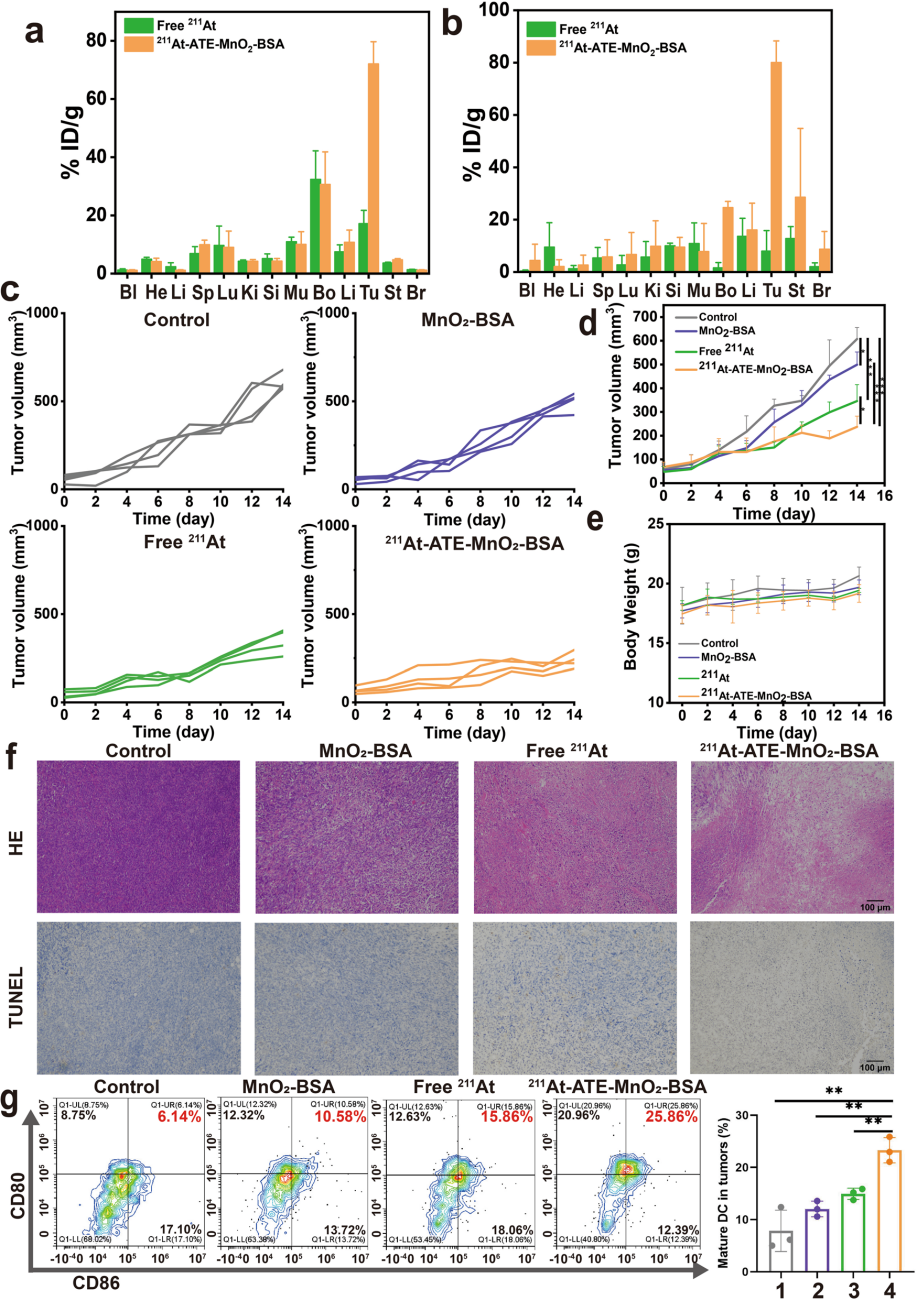
In vivo biodistribution and TAT/CDT based on ^211^At-ATE-MnO_2_-BSA. **(a-b)** Biodistribution of free ^211^At, ^211^At-ATE-MnO_2_-BSA in 4 T1 **(a)** and CT26 **(b)** mice at 12 h post i.t. injection. Error bars represent mean ± standard deviation (s.d.) (*n* = 3). **(c)** Tumor growth curves of each mouse in different groups. **(d)** Time-dependent tumor volume variations of 4 T1 tumor-bearing mice experiencing corresponding treatments in different groups. Error bars represent mean ± s.d. (*n* = 4). **(e)** Body weight variations of 4 T1 tumor-bearing Balb/c mice during treatment. Error bars represent mean ± s.d. (*n* = 4). **(f)** Optical microscopic images of H&E and TUNEL-stained tumor sections in different treatment groups. **(g)** FACS plots and statistical data of DC maturation induced by ^211^At-ATE-MnO_2_-BSA on mice bearing CT26 tumors. Tumors were collected 3 days after treatments and assessed by flow cytometry after stain with CD11c, CD80 and CD86. Error bars represent mean ± s.d. (*n* = 3). Note, 1: Control, 2:MnO_2_-BSA, 3: free ^211^At, 4: ^211^At-ATE-MnO_2_-BSA. *P* values were calculated by t-test (*P<0.05, **P < 0.01 and ***P < 0.001)

Inspired by the excellent therapeutic results in vitro, we explored the distribution of ^211^At-ATE-MnO_2_-BSA radioimmunotherapy promoters in 4 T1 tumor bearing mice and CT26 tumor bearing mice models, respectively. Compared to the free ^211^At group, the ^211^At-ATE-MnO_2_-BSA group exhibits high tumor retention and the accumulation in tumor remains high after 12 h post-injection (Fig. 2a and b), ensuring the subsequent anti-tumor outcomes. To further evaluate the therapeutic efficacy of astatine-211-labelled MnO_2_-BSA in vivo, the murine breast 4 T1 tumors with poor immunogenicity were divided into four groups randomly (*n* = 4), including: control, MnO_2_-BSA, free ^211^At (15 μCi) and ^211^At -ATE-MnO_2_-BSA (15 μCi). After 14 days post-treatment, the tumor volume in the control group grows rapidly. In contrast, the treatments in both MnO_2_-BSA group and free ^211^At group inhibit tumor growth to some certain extent via the GSH depletion-enhanced CDT and TAT, respectively. Once the two anti-tumor actions are integrated into ^211^At-ATE-MnO_2_-BSA radioimmunotherapy promoters, the most potent anti-tumor activity is reached, resulting in the largest inhibition rate of tumor growth (Fig. 2c and d, Table S1). Meanwhile, no negligible changes were found in the body weight after ^211^At -ATE-MnO_2_-BSA treatment, indicating good biocompatibility (Fig. 2e). Furthermore, to explore the mechanisms that cause the different therapeutic effects, hematoxylin–eosin (H&E) staining and terminal deoxynucleotidyl transferase dUTP nick end labeling (TUNEL) staining were performed. We find that ^211^At-ATE-MnO_2_-BSA radioimmunotherapy promoters acquires the highest cell damage/apoptosis, including chromatic agglutination, karyopyknosis, and nuclear fragmentation in hematoxylin-eosin (H&E) and brown stain in the TUNEL assay (Fig. 2f).

### The immune activation tests

To explore the principles of the TAT and CDT combined effects in enhancing above anti-tumor outcomes, immune-related indexes were explored. It has been reported that radiotherapy could trigger immune responses by releasing tumor-associated antigens. Dendritic cells (DCs) played a critical role both in innate and adaptive immune responses through engulfing and processing antigens and presenting them to activate T cells [37]. Thus, the co-stimulatory molecules CD80/CD86 which are the representative markers for DCs maturation, are detected by flow cytometry (FCM) [38]. Tumor tissues were collected from CT26 tumor-bearing mice after 3 days post-various treatments (MnO_2_-BSA, free ^211^At, ^211^At -ATE-MnO_2_-BSA, *n* = 3). Free ^211^At and ^211^At-ATE-MnO_2_-BSA could significantly promote the proportion of matured DCs, as compared to the untreated control group (Figs. 2g and S11). As a result, this result indicated that ^211^At labelled Mn-based radiosensitizer could serve as an immunostimulant to boost anti-tumor immune responses.

### Enhanced CDT-TAT-ICB tri-modal therapy for inhibiting metastasis

**Fig. 3:**
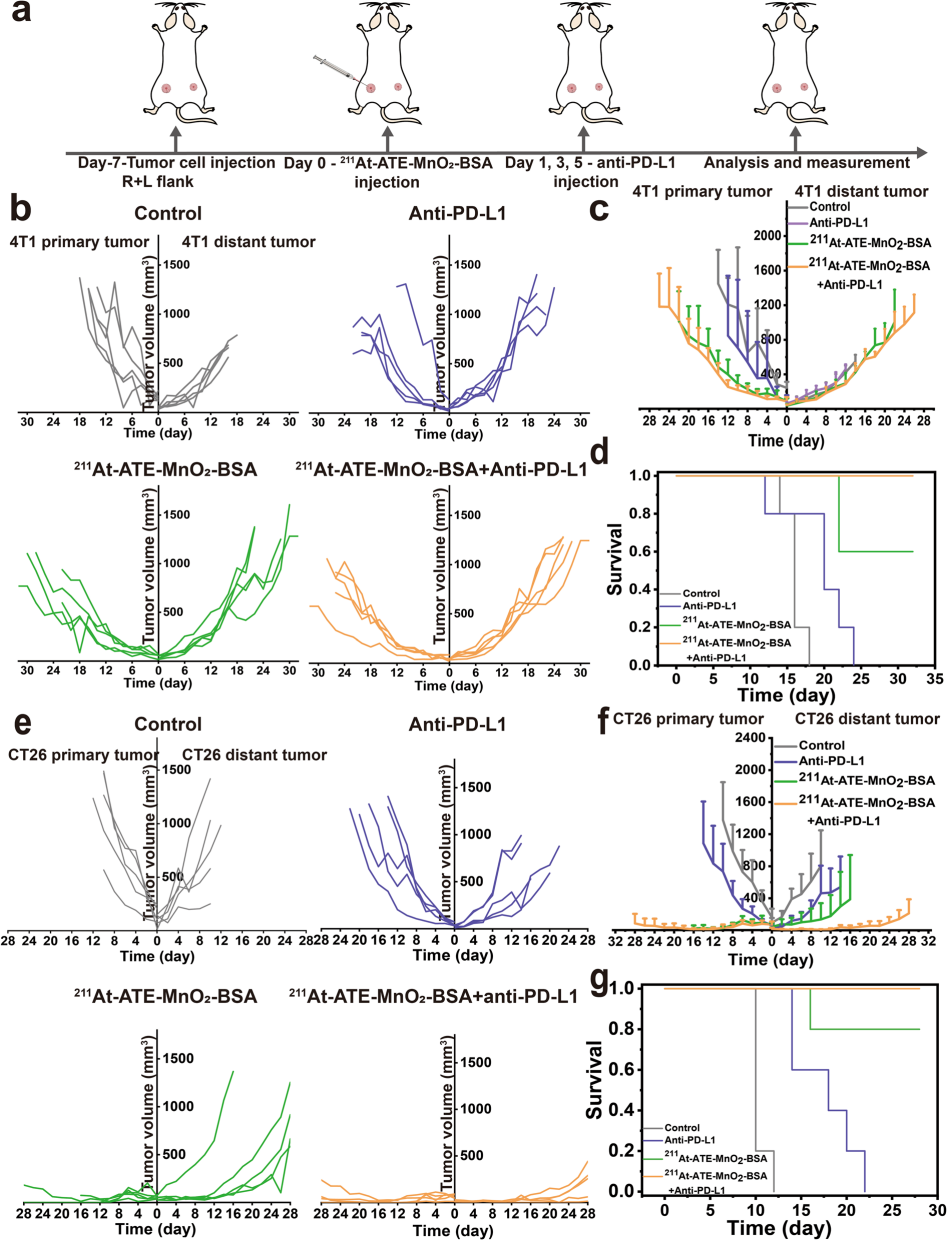
Distant tumor inhibition by tri-modal TAT/CDT/ICB combined therapy. **(a)** Schematic diagram of targeted alpha therapy plus anti-PD-L1 to suppress distant tumor growth. **(b-d)** Primary and distant tumor growth curves **(b-c)** and survival rates **(d)** of 4 T1 tumor-bearing mice after various treatments. Error bars represent mean ± s.d. (*n* = 5). **(e-g)** Primary and distant tumor growth curves **(e-f)** and survival rates. **(g)** of CT26 tumor-bearing mice after various treatments. Error bars represent mean ± s.d. (*n* = 5)

In previous studies, the PD-L1 expression on 4 T1 tumor cells was found to be too low to respond to anti-PD-L1 treatment. It has been documented that internal radioisotope therapy could regulate the expression of PD-L1 on tumors [39, 40]. Combining PD-L1 checkpoint blockade with internal radioisotope therapy will bring new hope for patients with metastases and relapse [41]. To validate the therapeutic efficiency of ^211^At-ATE-MnO_2_-BSA combined with PD-L1 blockade in metastasis tumors, the bilaterally 4 T1-bearing mice or CT26-bearing mice were adopted. 4 T1 (or CT26) cells inoculated in left flank of mouse were regarded as the primary tumor, the second tumor was inoculated in the right flank to mimic metastatic tumor. The experimental procedure is shown in Fig. 3a. For 4 T1 tumors, we observe that the tumors on both sides in untreated group grow rapidly at an uncontrollable rate. Compared with untreated group, the anti-PD-L1 group delays the tumor growth, but all the mice die within 24 days, resulting in the low survival rate. Intriguingly, the tumor growths in ^211^At-ATE-MnO_2_-BSA group and ^211^At-ATE-MnO_2_-BSA plus anti-PD-L1 group are also obviously inhibited, and concurrently the survival time is significantly prolonged, as evidenced in Fig. 3b-d. Consistent with the 4 T1 tumor model, ^211^At-ATE-MnO_2_-BSA plus anti-PD-L1 treatment not only completely destroys the local CT26 tumors, but also greatly inhibits the growth of distant tumors (Fig. 3e and f, and Figs. S7 and S8). Notably, the TAT/CDT/ICB tri-modal synergistic group shows a considerably-prolonged survival rate than other groups (Fig. 3g). As well, all the tumor-bearing mice have no abnormal body-weight changes in this synergistic group, and no pathological changes and no liver and kidney function changes are found in the major organs, indicating the high therapeutic biosafety of IRT/ICB treatment (Figs. S6, S9 and S10).

### Mechanism of systematic antitumor immune responses

**Fig. 4:**
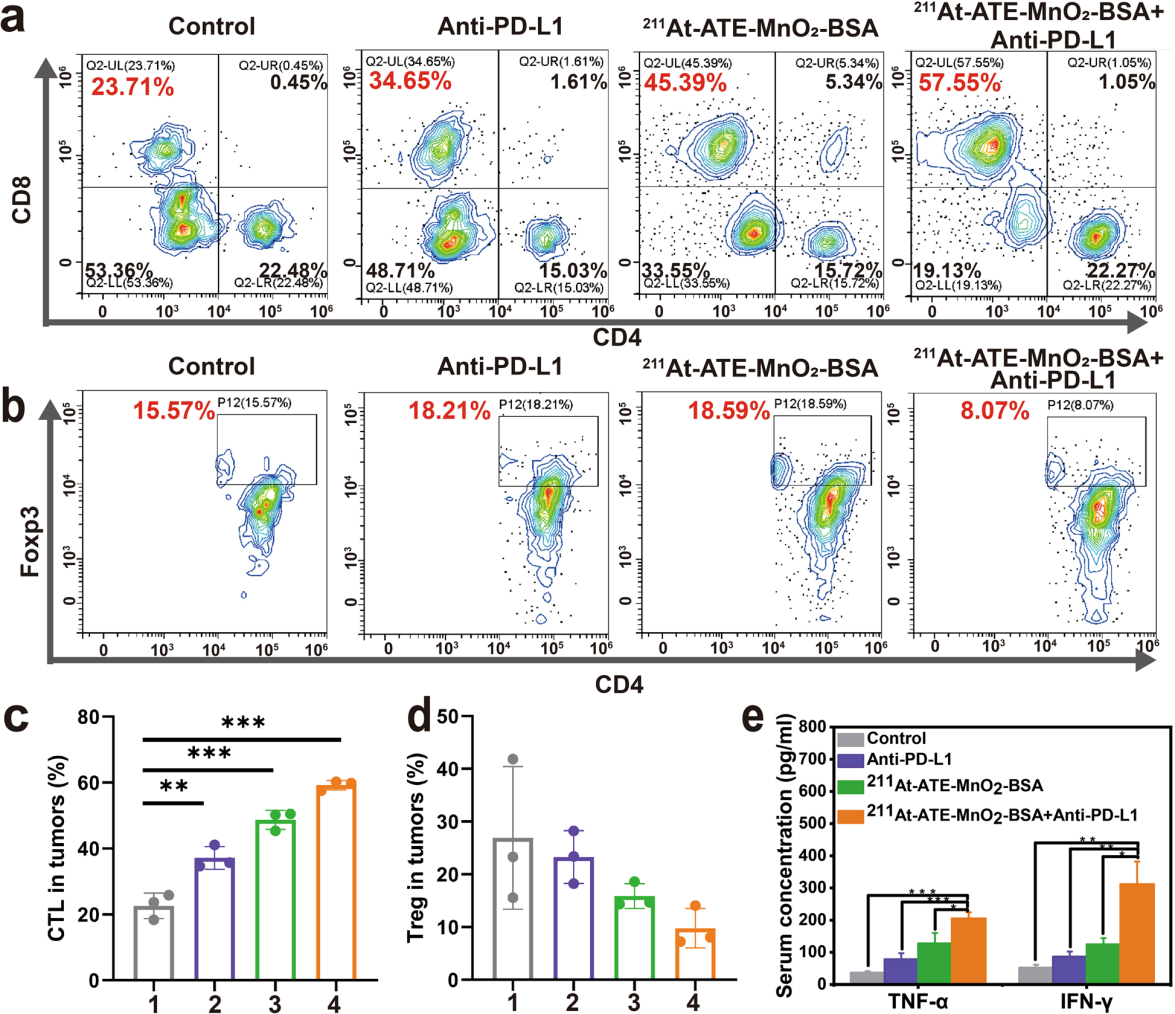
Activated immune response tests by TAT/CDT/ICB. **(a-b)** Representative flow cytometry plots showing T cells **(a)** and Treg cells **(b)** in the distant tumors from different groups 10 days post-treatment (CT26 tumor model). **(c)** Proportions of tumor-infiltrating CD8^+^ killer T cells among CD3^+^ cells (CT26 tumor model). Error bars represent mean ± s.d. (*n* = 3). **(d)** Proportions of Treg cells in tumor. Error bars represent mean ± s.d. (*n* = 3). **(e)** TNF-α level and interferon-γ (IFN-γ) level in mice sera post-various treatments (CT26 tumor model). Note, 1: Control, 2: Anti-PD-L1, 3: ^211^At-ATE-MnO_2_-BSA, 4: ^211^At-ATE-MnO_2_-BSA + Anti-PD-L1. P values were calculated by t-test (*P<0.05, **P < 0.01 and ***P < 0.001)

To understand the underlying mechanism of the antitumor effects triggered by ^211^At -ATE-MnO_2_-BSA plus anti-PD-L1, immune cells in the distant tumors were assessed on the 10th day after the first treatment on the bilaterally CT26 tumors-bearing mouse model. Cytotoxic T lymphocytes (CTLs) (CD3^+^CD4^−^CD8^+^) cells are essential for the tumor immunotherapy [42]. Results show that the proportion of intratumoral CD8^+^ T cells is increased in the distant tumors after 10 days post-treatment with ^211^At-ATE-MnO_2_-BSA plus anti-PD-L1 (Fig. 4a and c), which is consistent with previous studies [23, 43]. Concurrently, immunosuppressive lymphocytes including regulatory T cells (Tregs) have no obvious changes in the distant tumors (Fig. 4b and d). Furthermore, tumor necrosis factor-alpha (TNF-α) and interferon gamma (IFN-γ) that are important markers of cellular antitumor immunity and involve in the cytotoxic functions of CTLs are increased significantly (Fig. 4e) [44]. These results suggest the activated immune responses by the synergistic treatment effects of TAT, CDT and ICB in such radioimmunotherapy promoters.

### Long-term immune-memory effects for repressing relapse

**Fig. 5:**
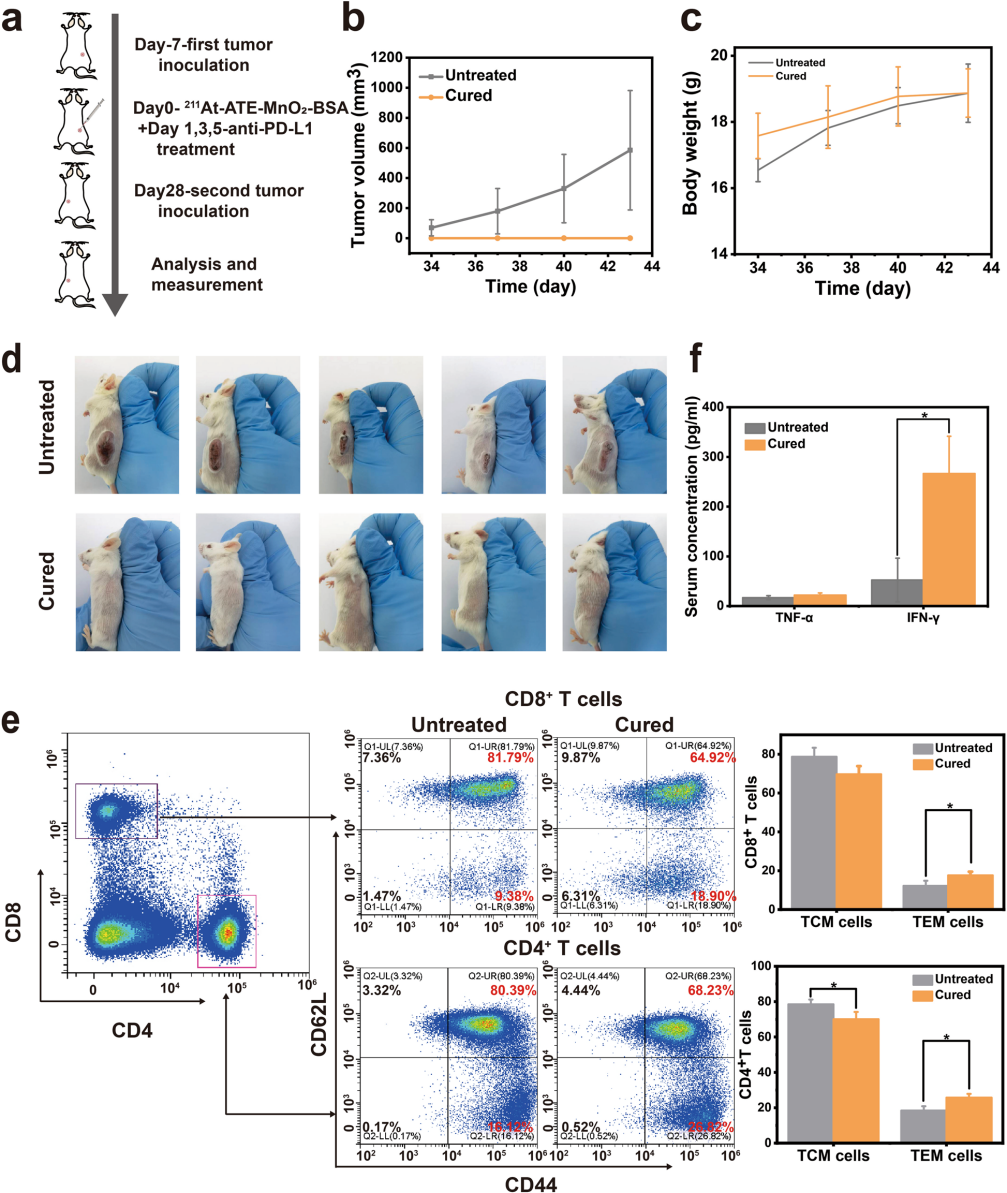
TAT/CDT/ICB trimodal treatment for inhibiting tumor cell rechallenge. **(a)** Schematic illustration of the combination therapy to generate anticancer immune memory and inhibition of cancer recurrence. **(b)** Growth curves of the rechallenged tumors after different treatments. Data are mean ± s.d. (*n* = 5). **(c)** Body weight of balb-c mice. Data are expressed as mean ± SD (*n* = 5). **(d)** Photographs of ^211^At-ATE-MnO_2_-BSA + anti-PD-L1 cured mice and untreated mice receiving CT26 tumor cells. **(e)** Representative flow cytometric analysis images and corresponding quantification results of the percentage of central memory T cells (TCM) and effector memory T cells (TEM) of splenic lymphocytes of different groups 15 days after CT26 tumor cell rechallenge (*n* = 3). **(f)** Expression levels of serum cytokines including TNF-α and IFN-γ from mice after the secondary tumor rechallenge (*n* = 3). P values were calculated by t-test (*P<0.05, **P < 0.01 and ***P < 0.001)

As a hallmark of adaptive immunity, immunological memory response is able to protect organisms from the second attack of pathogen infection, including cancer cells [45, 46]. To evaluate whether the tri-modal TAT/CDT/ICB combined treatment could induce an immune memory effect, CT26 cells were inoculated 28 d on the opposite (left) side after removing the initial CT26 tumors (right) by this combination treatment, as illustrated in Fig. 5a. At the same time, five mice inoculated with equal numbers of cells on the left without treatment were set as the control group. The tumors in control group grow rapidly at an uncontrolled speed. In contrast, a remarkable and appealing result is obtained that no rechallenged tumor on the left side is detected in all mice after their initial tumors on the right side are completely inhibited by ^211^At -ATE-MnO_2_-BSA plus anti-PD-L1 therapy (Fig. 5b and d). Besides, no obvious changes are found in the body weight (Fig. 5c). Taken all together, these results clearly reveal that the long-term immune memory effect enables the treated mice to resist tumor relapse.

To explore the mechanisms of immune memory generated by the combined therapy, spleens were collected on day 15th day post-rechallenge, and the memory T cells were evaluated by flow cytometry. It is known that the memory T cells can be divided into central memory T cells (TCM, CD3^+^CD8^+^CD62L^+^CD44^+^) and effector memory T cells (TEM, CD3^+^CD8^+^CD62L^−^CD44^+^) [47]. TCM fails to provide protection at the beginning of antigen-stimulation, and they can exert the killing effects only if they undergo a series of processes such as expansion and differentiation [48]. Differing from TCM cells, TEM cells could instantly induce protection through secreting important cytokines such as TNF-α and IFN-γ after meeting the same pathogen, which are identified as the direct executor of immunotherapy [49]. Interestingly, the proportions of TEM in both CD8^+^ and CD4^+^ T cell populations are significantly increased after ^211^At-ATE-MnO_2_-BSA plus anti-PD-L1 therapy, whereas the percentage of TCM is decreased (Fig. 5e). Moreover, the level of IFN-γ was obviously up-regulated, suggesting the robust antitumor immune responses generated by the combination therapy strategy (Fig. 5f).

## Discussion

It has been documented that IRT based on β-particles is widely used in clinics, which not only kills tumor cells through the direct killing effect, but also can elicit immune responses to attack the tumor cells to some extent [3]. However, it usually causes physiological toxicity and radiation resistance due to the existence of a hypoxic tumor microenvironment [4, 5]. Moreover, the low production efficiency of ROS induces poor immune responses, which is not sufficient to repress tumor metastasis and relapse.

In this report, α-particles with the advantages of high linear energy transfer (> 50 keV/μm) were developed based on MnO_2_-BSA, leading to more DNA double-strand injures and relative biological efficiency elevation with a lower radiation dose. The cyclotron-produced ^211^At address currently inadequate supplies of medically useful α-emitters. Thus, ^211^At is regarded to be the most suitable candidate for TAT, which is sufficient for labeling process, quality control, and medical application. In addition, the short half-life won’t cause medical problems by long-lived daughters of α-emitting radionuclide.

CDT is an emerging therapeutic strategy by utilizing Fenton or Fenton-like reactions to modulate the immunogenic tumor microenvironment and enhance ICB. It was reported that Fe-based CDT could trigger tumor-associated antigens release and further allowed to be taken up and captured by DCs，leading to systemic immune responses. However, CDT alone also fails to sufficiently activate immune responses, and it usually requires combined therapy such as radiotherapy. Fortunately, MnO_2_-BSA in our radioimmunotherapy promoters could trigger Fenton-like reactions to deplete H_2_O_2_ and GSH and realize redox balance disruption-enhanced CDT for producing ROS. The enhanced CDT synergized with TAT to activate robust immune responses to repress tumor progression including primary and distant tumors.

ICB is widely used in a broad range of cancers, such as non-small cell lung cancer [50], clear cell renal cell carcinoma [51], breast cancer [52], melanoma [53], and head and neck cancer [54]. However, it seems to be effective in only 20% of cancer patients due to the ITM and immune-desert cold tumors with low mutation burdens, low neoantigen burden, and low level of effector T cells [16]. Inspiringly, besides activating systematic immune responses, the massive ROS production in the TAT/CDT combined therapy also favor ITM liberation, and they further united with ICB to significantly delay tumor growth and relapse via activating long-term immune memory effects on the challenge model, which means such radioimmunotherapy promoters could serve as tumor vaccines of non-small cell lung cancer, clear cell renal cell carcinoma, breast cancer, colon cancer, and others.

## Conclusions

In summary, Mn-based radioimmunotherapy promoters with high radiolabeling stability were designed and developed to promote the therapeutic efficacy of ^211^At both in vitro and in vivo through the TAT/CDT synergistic effects. It is noteworthy that such TAT-CDT could promote DC maturity and boost systematic anticancer immune responses. Besides, with the help of anti-PD-L1 blockade, distant tumors were obviously inhibited by cooperative TAT-CDT-ICB both in bilaterally 4 T1 and CT26-bearing mice. The increasing of CD45^+^ leucocytes and CTL, and the reduction of Tregs as well as the rising cytokine secretion prove that the enhanced immunotherapy. Excitingly, the trimodal TAT/CDT/ICB combined therapy strategy could induce long-term immunological memory to resist tumor rechallenge owing to the generation of T memory cells. For clinical translation, this therapeutic modality might provide more opportunities for patients with metastatic tumors and prevent tumor relapse.

## Supplementary Information

**Additional file 1.**

## Data Availability

Not applicable.

## Abbreviations

TAT: Targeted alpha therapy
CDT: Chemodynamic therapy
BSA: Bovine serum albumin
DCs: Dendritic cells
RT: Radiotherapy
IRT: Internal radiotherapy
EBRT: External-beam radiotherapy
ITM: Immunosuppressive tumor microenvironment
ICB: Immune checkpoint blocking
ATE: N-succinimidyl 3-trimethylstannyl-benzoate
ROS: Reactive oxygen species
TAAs: Tumor-associated antigens
CTLs: Cytotoxic T lymphocytes
DLS: Dynamic light scattering
MB: Methylene blue
DMPO: 5,5-dimethyl-1-pyrroline N-oxide
HE: Hematoxylin–eosin
TUNEL: Terminal deoxynucleotidyl transferase dUTP nick end labeling
FCM: Flow cytometry
Tregs: Regulatory T cells
TNF-α: Tumor necrosis factor-alpha
IFN-γ: Interferon-gamma
TCM: Central memory T cells
TEM: Effector memory T cells
MnCl_2_·4H_2_O: Manganese chloride tetrahydrate
NaOH: Sodium hydroxide
FBS: Fetal bovine serum
CCK-8: Cell counting kit-8
μCi: Micro Curie
