# Supplementary material for: Alpha radionuclide-chelated radioimmunotherapy promoters enable local radiotherapy/chemodynamic therapy to discourage cancer progression
Source: Biomater Res. 2022 Sep 8;26:44. doi: 10.1186/s40824-022-00290-6 (PMC9461185; doi:10.1186/s40824-022-00290-6)
Supplement: Supplementary file 1 — Additional file 1. [file 40824_2022_290_MOESM1_ESM.docx]

Supporting Information

**Alpha Radionuclide-Chelated** **Radioimmunotherapy Promoters Enable Local Radiotherapy/Chemodynamic Therapy to Discourage Cancer Progression**

*Jiajia Zhang,^1,2,3,#^ Feize Li, ^4,#^ Yuzhen Yin,^1,2,#^ Ning Liu,^4^ Mengqin Zhu,^1,2^ Han Zhang,^1,2,^ Weihao Liu,^4^ Mengdie Yang,^1,2,^ Shanshan Qin,^1,2^ Xin Fan,^1,2^ Yuanyou Yang,^4,^* Kun Zhang,^2,3,^* Fei Yu^1,2,^**

^1^ Department of Nuclear Medicine, Shanghai Tenth People's Hospital, Tongji University School of Medicine, No. 301 Yan-chang-zhong Road, Shanghai 200072, P. R. China

^2^ Institute of Nuclear Medicine, Tongji University School of Medicine, No. 301 Yan-chang-zhong Road, Shanghai 200072, P. R. China

^3^ Department of Medical Ultrasound and Central Laboratory, Ultrasound Research and Education Institute, Shanghai Tenth People’s Hospital, Tongji University School of Medicine, No. 301 Yan-chang-zhong Road, Shanghai 200072, P. R. China

^4^ Key Laboratory of Radiation Physics and Technology of the Ministry of Education, Institute of Nuclear Science and Technology, Sichuan University, Chengdu 610064,

PR China

*Correspondence to: Fei Yu ([yufei_021@163.com](mailto:yufei_021@163.com)), Kun Zhang ([zhang1986kun@126.com](mailto:zhang1986kun@126.com)) and Yuanyou Yang （[yangyy@scu.edu.cn](mailto:yangyy@scu.edu.cn)）

^#^ The authors contributed equally to this work.

**
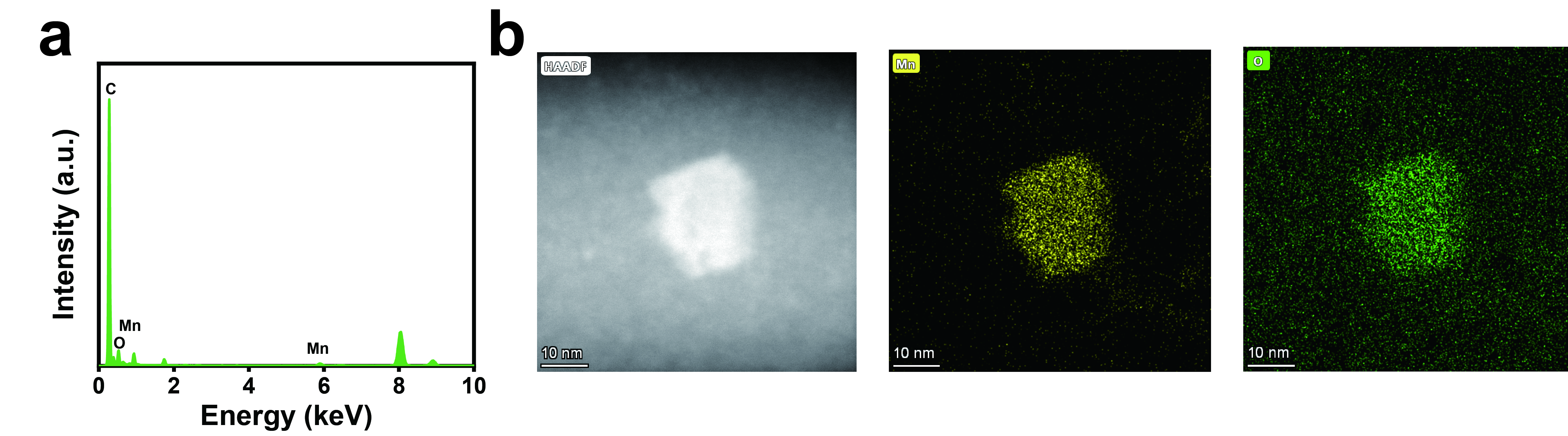
**

**Fig. S1** (a) EDS of MnO_2_-BSA. (b) high-angle annular dark field image and corresponding elemental-mapping images of MnO_2_-BSA.

**
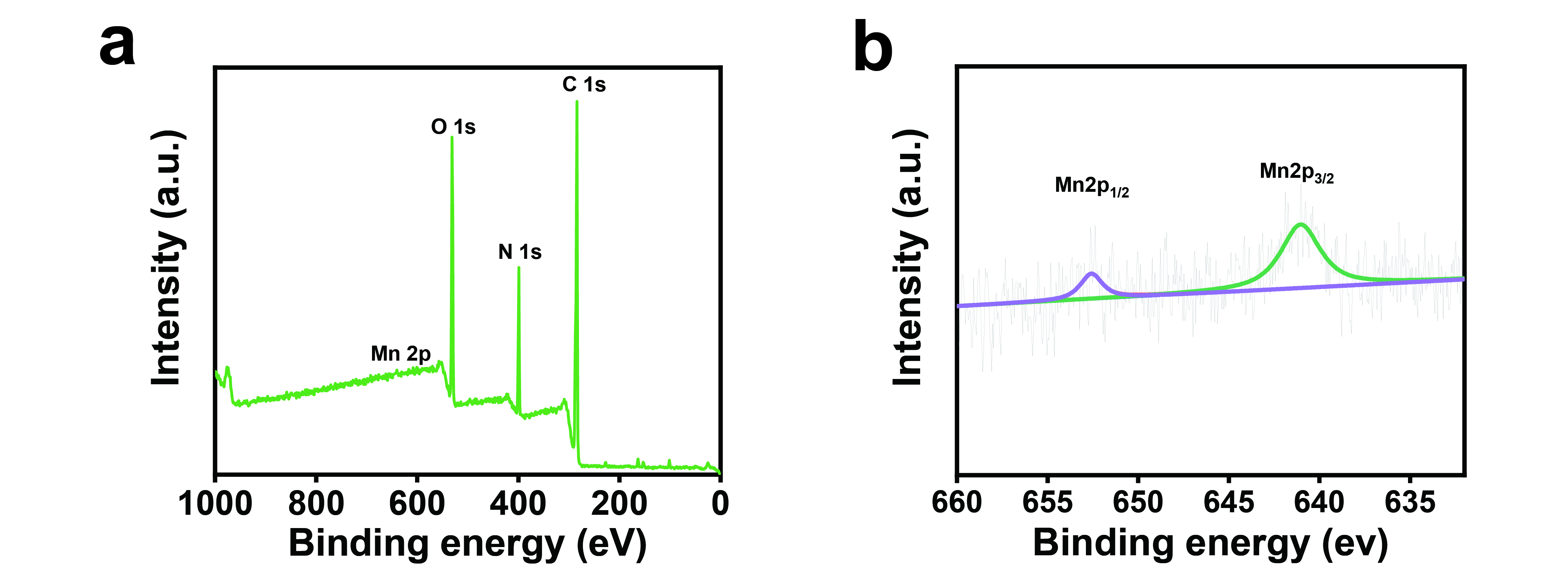
**

**Fig. S2** (a) XPS survey spectra and (b) high resolution Mn2p XPS spectra of MnO_2_-BSA.

**
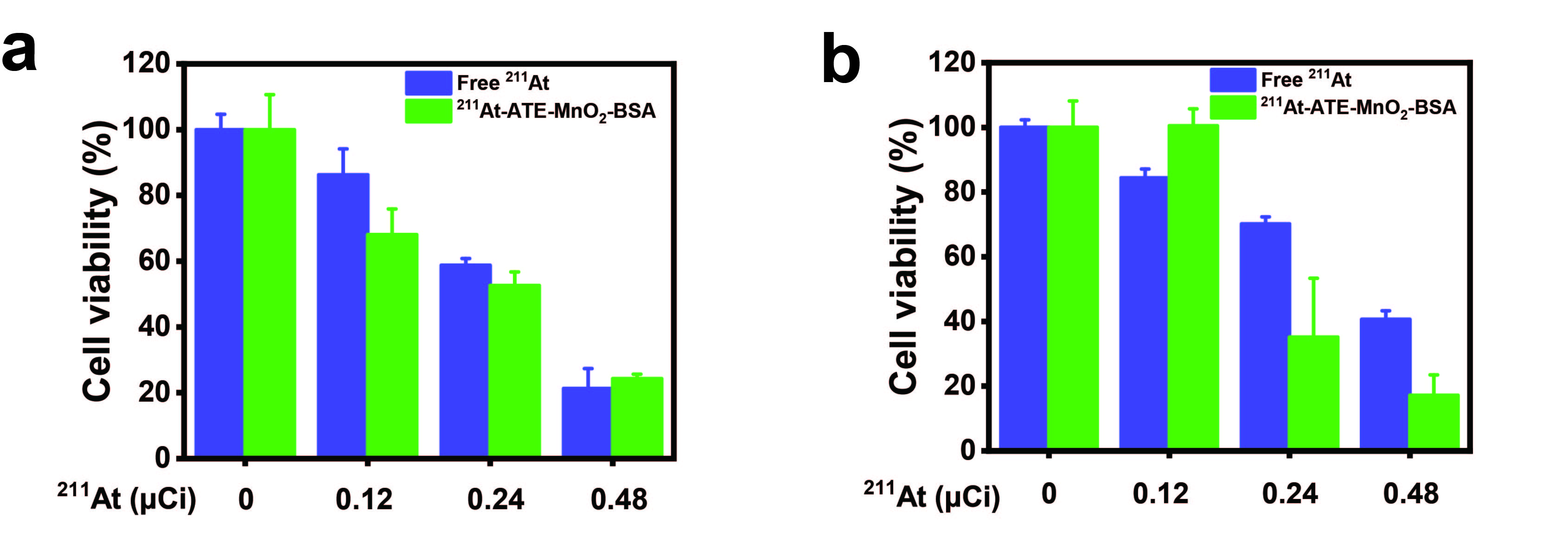
**

**Fig. S3 (a-b)** Cytotoxicity of free ^211^At and ^211^At-ATE-MnO_2_-BSA against 4T1 **(a)** and CT26 **(b)** cells at various concentrations at 12 h.

**
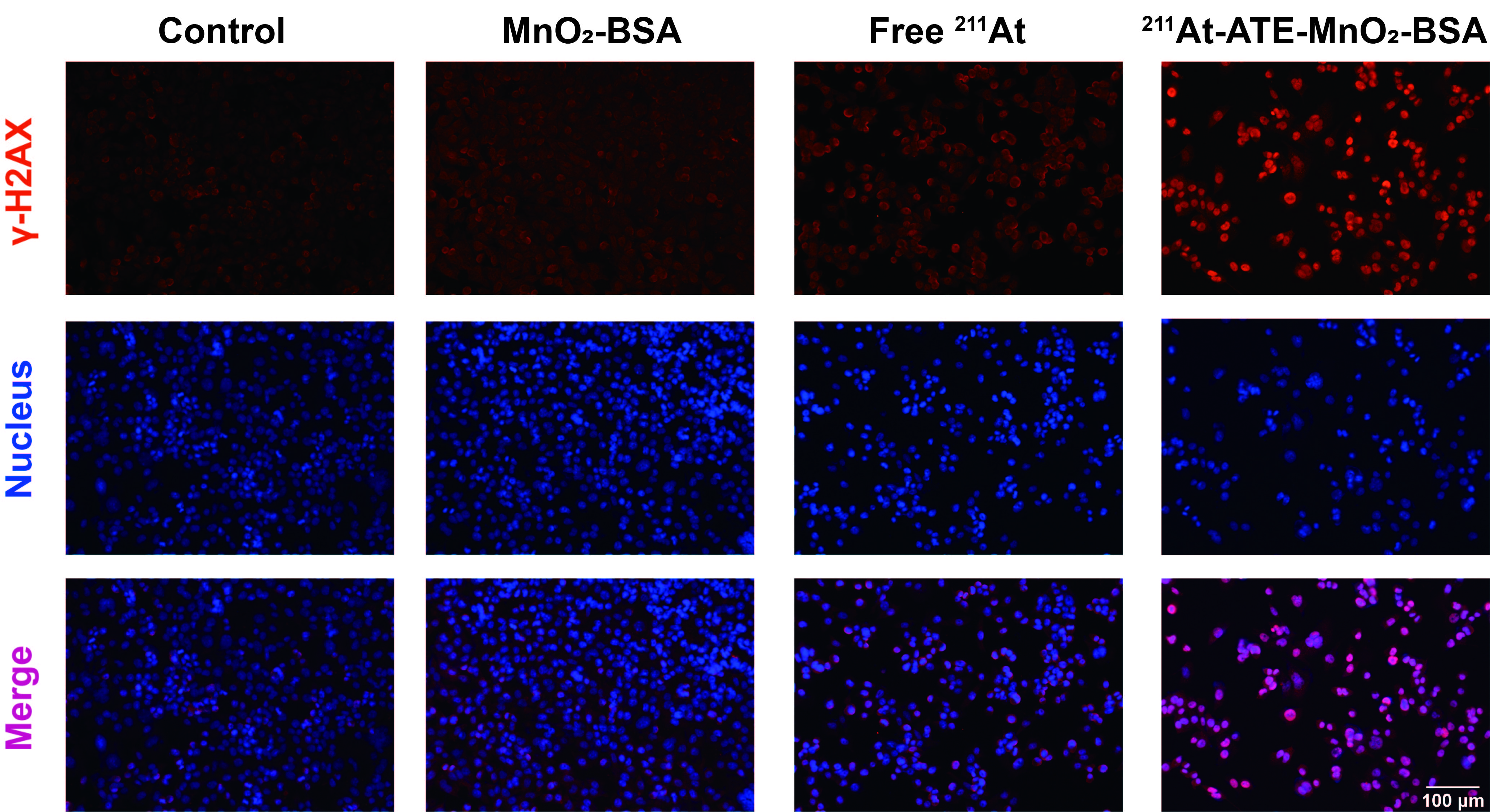
**

**Fig. S4** γ-H2AX fluorescence images (blue: DAPI, red: γ-H2AX) of 4T1 cells 12 h after different treatments (Control, MnO_2_-BSA, free ^211^At and ^211^At-ATE-MnO_2_-BSA).


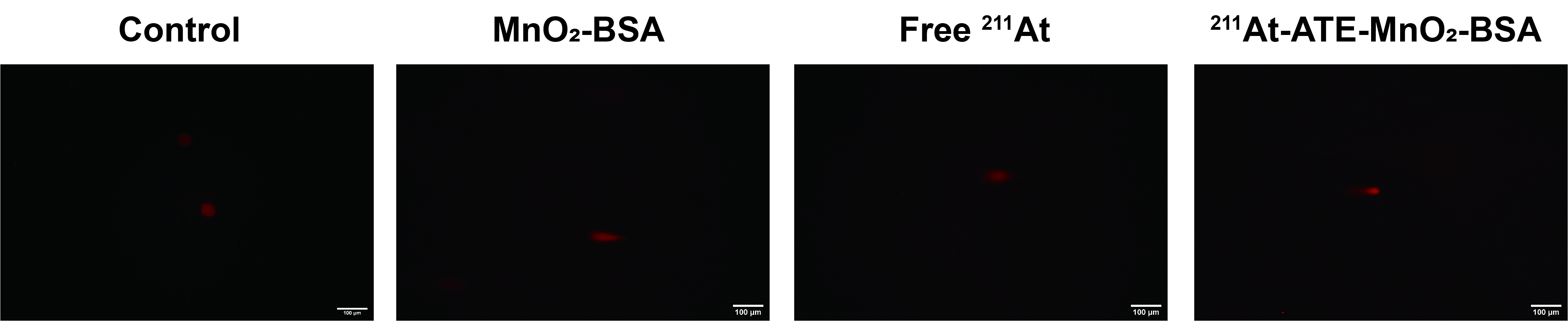


**Fig. S5 (a)** Comet assay to evaluate the DNA damage levels of 4T1 cells in various treatments. Quantification of **(b)** comet tailDNA, **(c)** tail length, and **(d)** tail moment of 4T1 cells in different treatment groups.

**
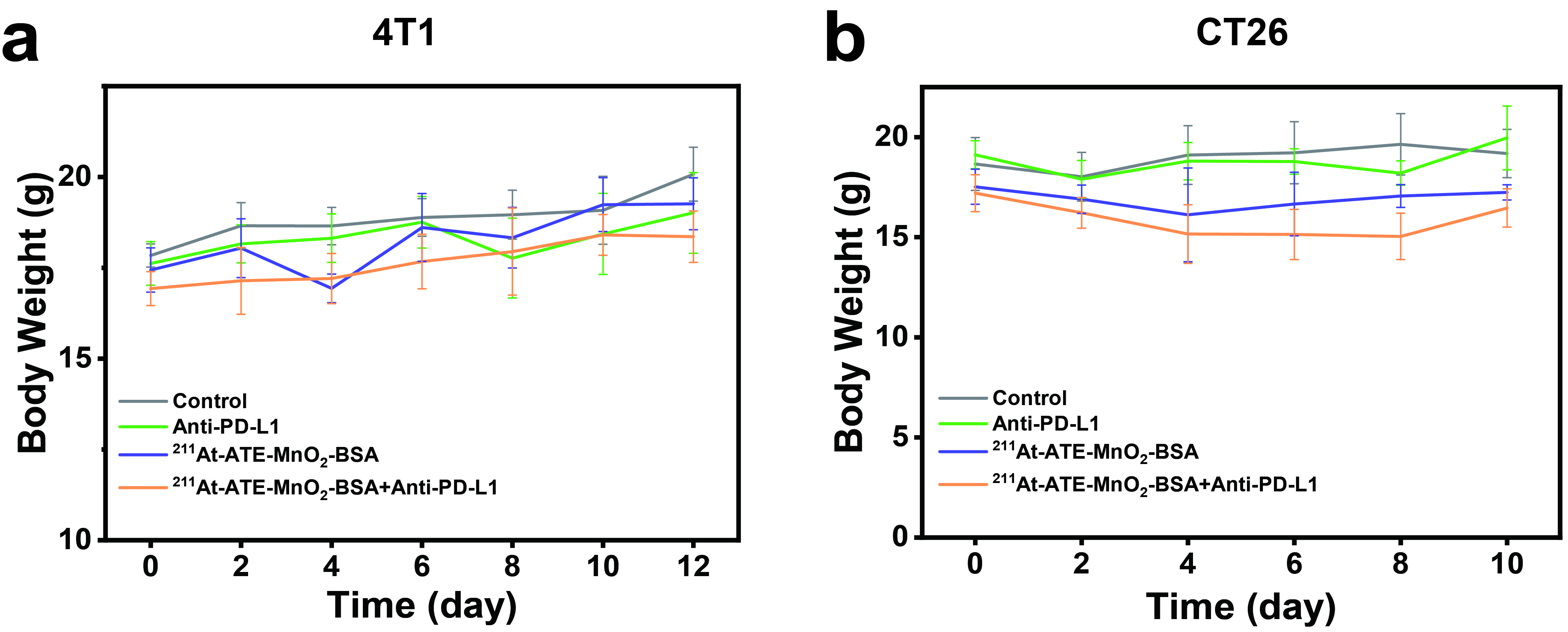
**

**Fig. S6 (a-b)** Body weight variations of bi-lateral 4T1 tumors-bearing Balb/c mice **(a)** and bi-lateral CT26 tumors-bearing Balb/c mice **(b)** during different treatments.

**
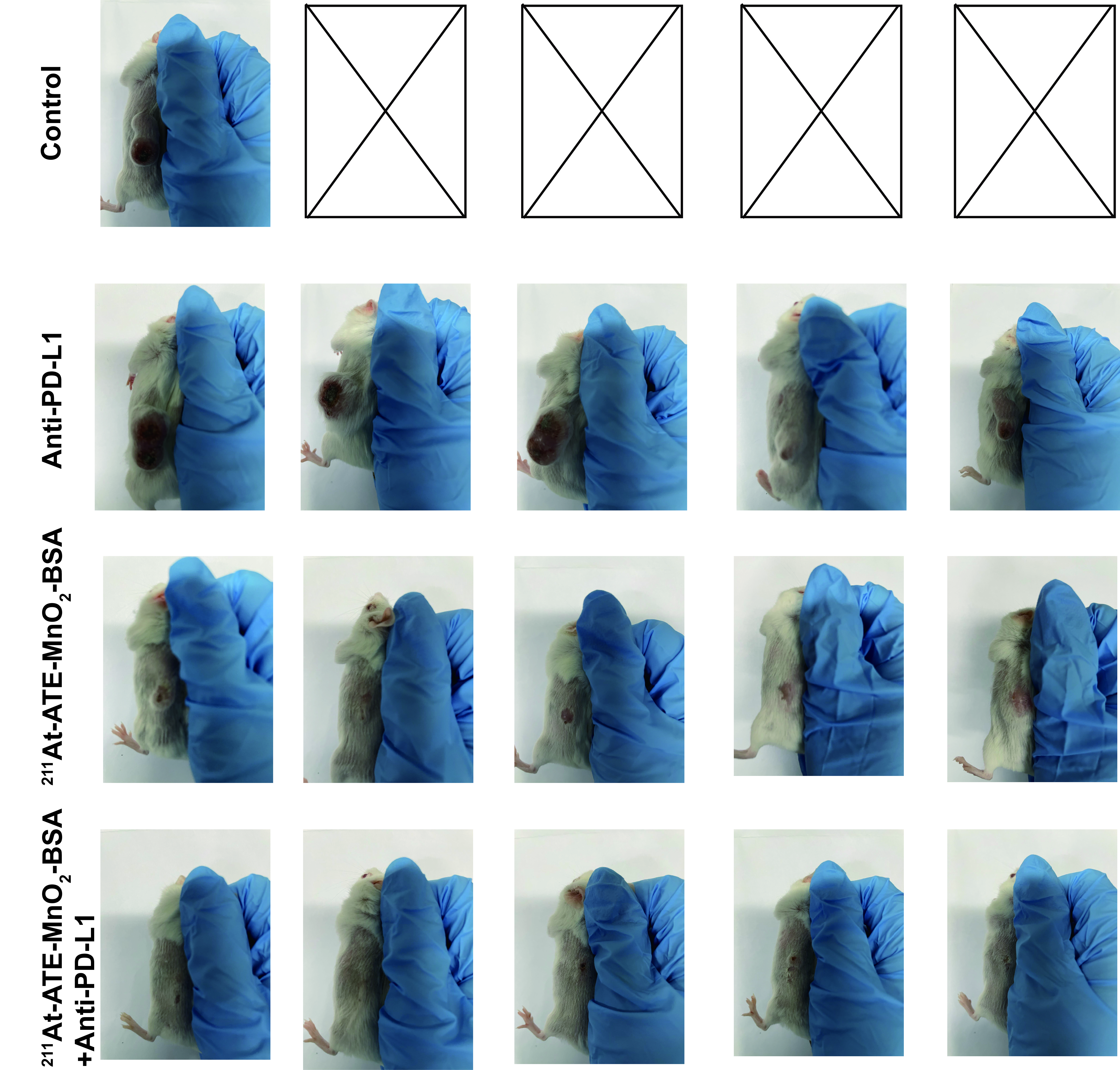
**

**Fig. S7** Digital photos of primary tumors implanted on bilateral CT26 tumor-bearing mice that experienced different treatments (day13).

**
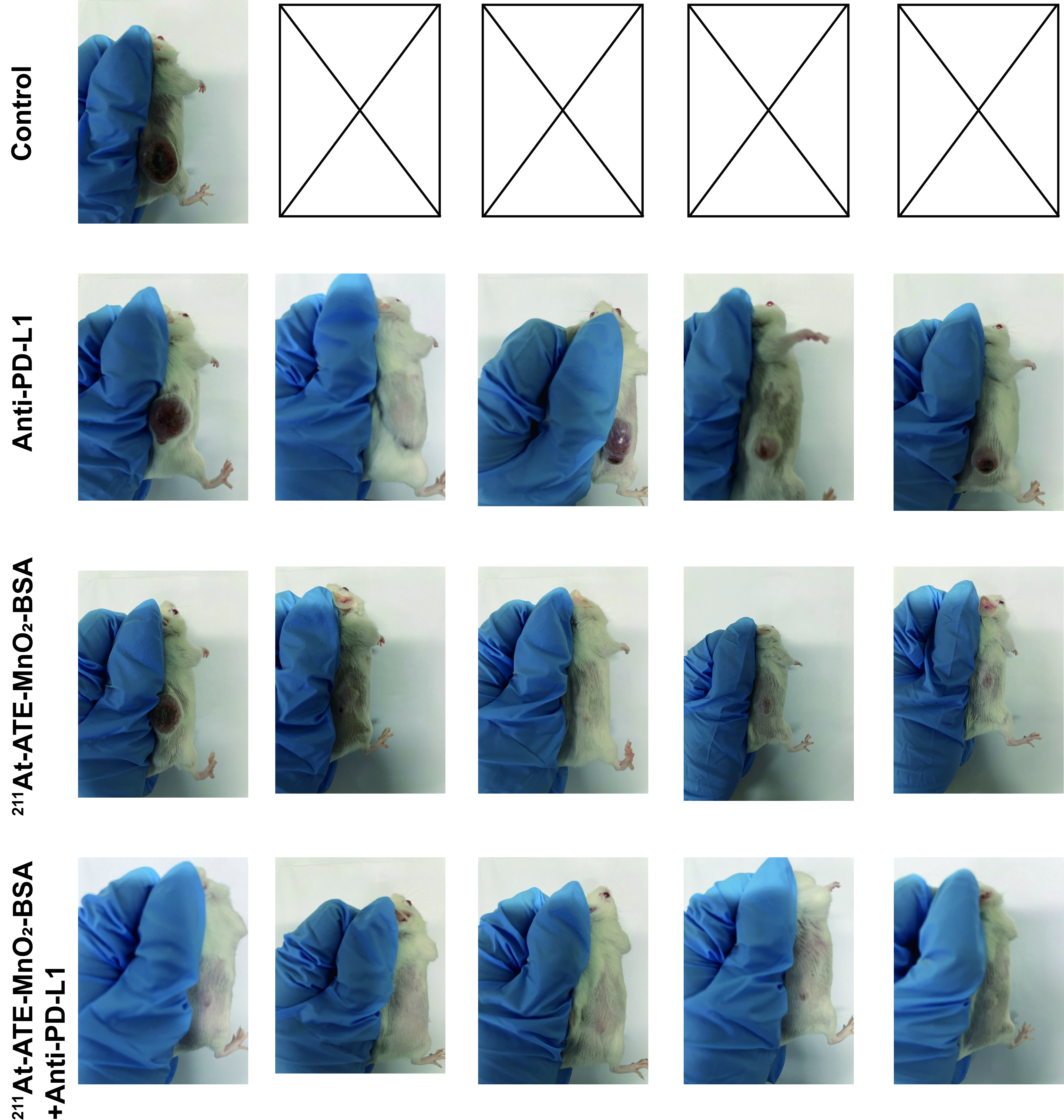
**

**Fig. S8** Digital photos of distant tumors implanted on bilateral CT26 tumor-bearing mice that experienced different treatments (day13).

**
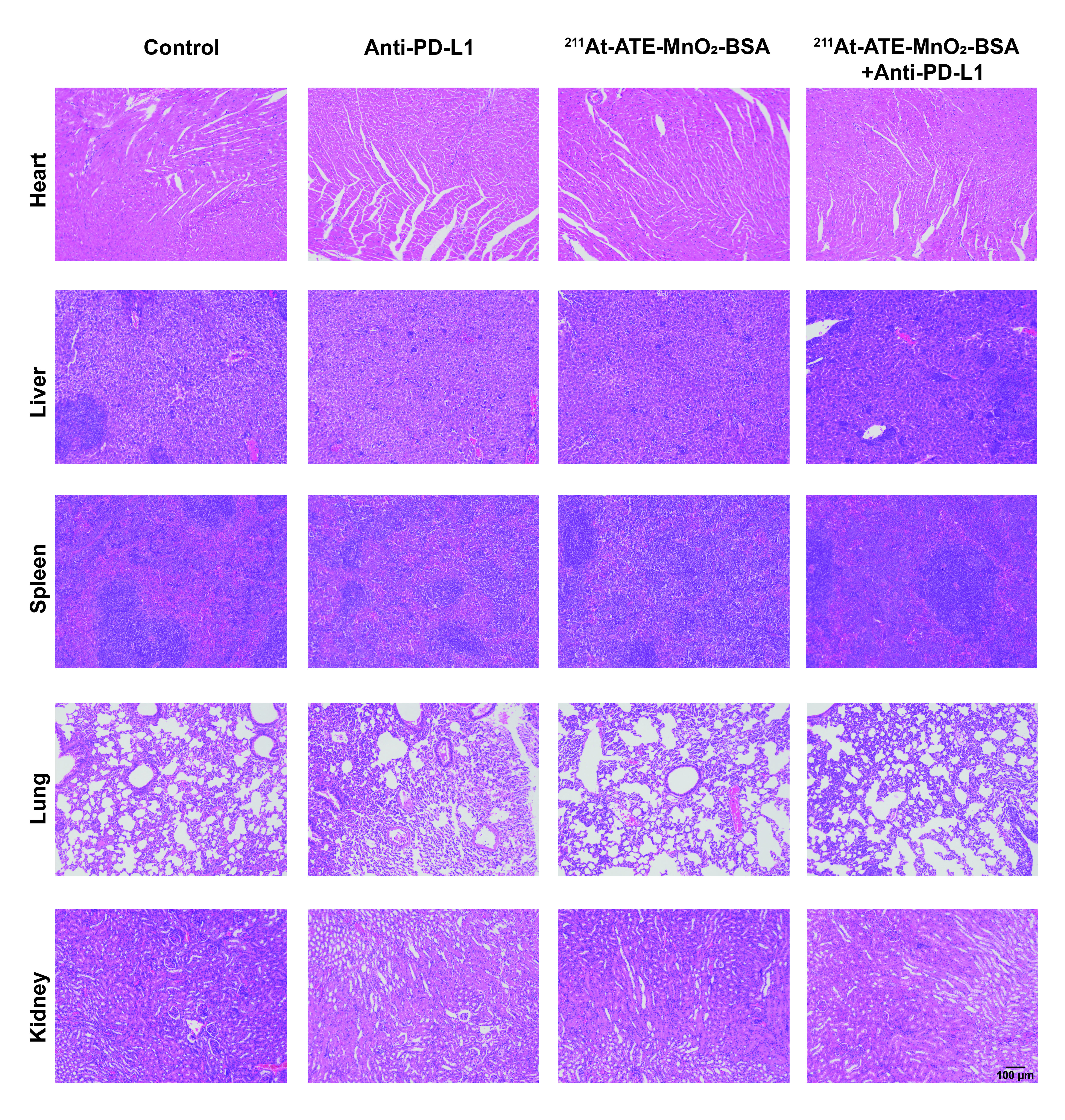
**

**Fig. S9** In vivo biosafety evaluation post-different treatments. H&E-stained for the major organs (*e.g.*, heart, liver, spleen, lung and kidney), and scale bar: 100 μm.

**
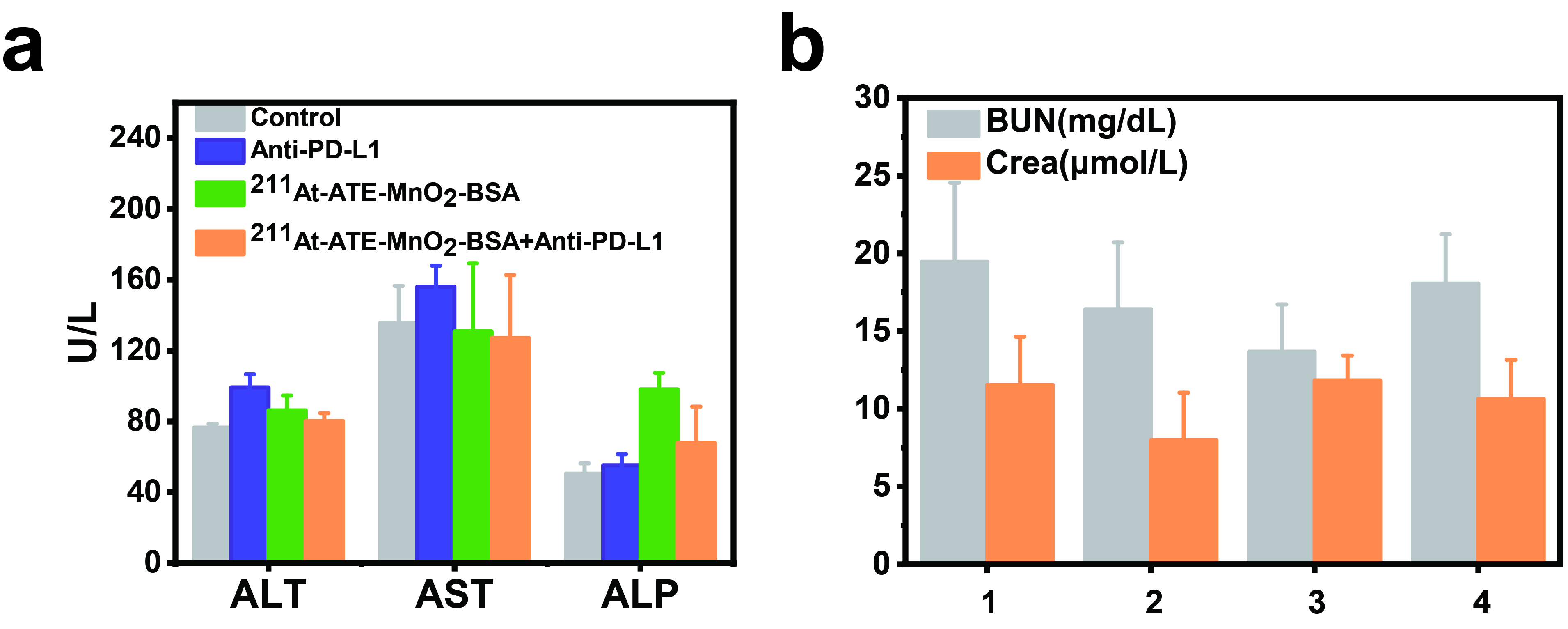
**

**Fig. S10** In vivo biosafety evaluation post-different treatments. Liver (*e.g.*, ALT, AST, ALP) and renal (BUN, Crea) function indexes analysis. Note: 1-4 represent Control, anti-PD-1, ^211^At-ATE-MnO_2_-BSA and ^211^At-ATE-MnO_2_-BSA+anti-PD-1 treatment, respectively.


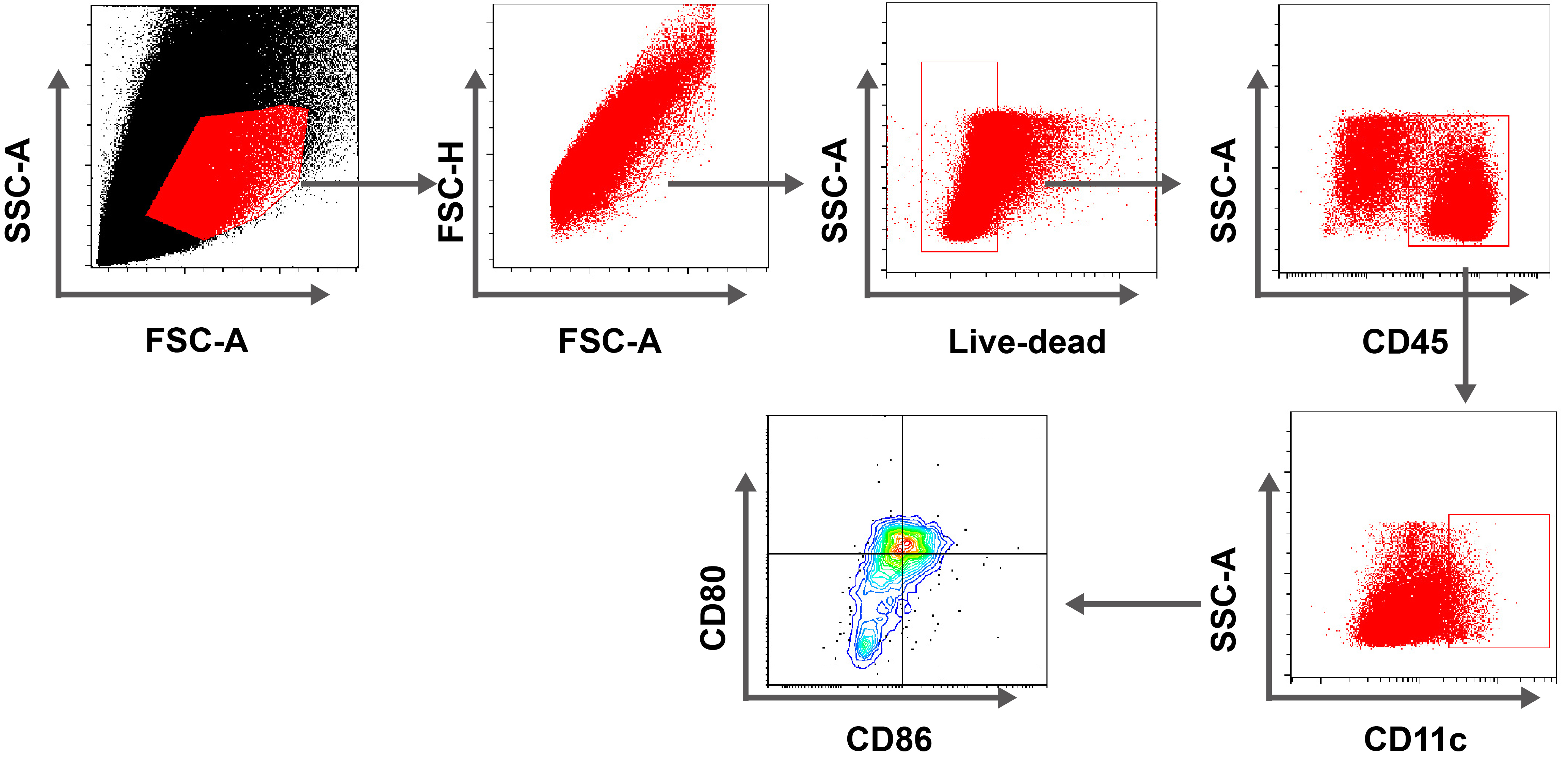


**Fig. S11** Gating strategy of dendritic cells.

**Table. S1** Tumor volume (mm^3^) of each mouse in different groups.

| **Time (Day)** | **Control group** | | | | **MnO_2_-BSA group** | | | | **p-value** |
| --- | --- | --- | --- | --- | --- | --- | --- | --- | --- |
| 0 | 27.9 | 81.6 | 67.2 | 54.4 | 64.0 | 29.7 | 53.4 | 68.8 | 0.799 |
| 2 | 20.3 | 103.7 | 103.7 | 92.5 | 62.1 | 42.8 | 74.0 | 75.6 | 0.474 |
| 4 | 99.1 | 123.8 | 144.7 | 189.1 | 163.2 | 98.8 | 141.3 | 52.9 | 0.448 |
| 6 | 274.2 | 130.9 | 194.6 | 265.9 | 140.5 | 104.4 | 171.0 | 172.8 | 0.113 |
| 8 | 313.6 | 312.0 | 368.0 | 312.1 | 334.6 | 213.2 | 257.3 | 220.5 | 0.065 |
| 10 | 342.1 | 365.6 | 362.0 | 318.5 | 376.5 | 257.3 | 382.9 | 298.4 | 0.594 |
| 12 | 416.0 | 385.3 | 604.7 | 569.9 | 427.1 | 413.5 | 449.2 | 454.1 | 0.337 |
| 14 | 573.2 | 594.1 | 583.2 | 678.9 | 514.0 | 421.6 | 542.7 | 518.9 | 0.024* |
|  | **Control group** | | | | **^211^At** | | | |  |
| 0 | 27.9 | 81.6 | 67.2 | 54.4 | 31.5 | 74.4 | 58.7 | 26.1 | 0.555 |
| 2 | 20.3 | 103.7 | 103.7 | 92.5 | 46.9 | 80.8 | 62.9 | 45.6 | 0.372 |
| 4 | 99.1 | 123.8 | 144.7 | 189.1 | 87.5 | 156.8 | 141.7 | 121.8 | 0.633 |
| 6 | 274.2 | 130.9 | 194.6 | 265.9 | 97.5 | 147.8 | 127.5 | 171.5 | 0.074 |
| 8 | 313.6 | 312.0 | 368.0 | 312.1 | 167.4 | 165.6 | 151.7 | 116.8 | ＜0.001*** |
| 10 | 342.1 | 365.6 | 362.0 | 318.5 | 249.7 | 257.3 | 235.4 | 213.2 | ＜0.001*** |
| 12 | 416.0 | 385.3 | 604.7 | 569.9 | 324.5 | 336.5 | 293.6 | 238.2 | 0.016* |
| 14 | 573.2 | 594.1 | 583.2 | 678.9 | 406.6 | 396.1 | 321.4 | 259.2 | ＜0.001*** |
|  | **Control group** | | | | **^211^At-ATE-MnO_2_-BSA** | | | |  |
| 0 | 27.9 | 81.6 | 67.2 | 54.4 | 96.3 | 65.6 | 47.7 | 63.0 | 0.523 |
| 2 | 20.3 | 103.7 | 103.7 | 92.5 | 130.1 | 91.0 | 58.2 | 72.0 | 0.769 |
| 4 | 99.1 | 123.8 | 144.7 | 189.1 | 209.5 | 130.0 | 79.5 | 106.9 | 0.828 |
| 6 | 274.2 | 130.9 | 194.6 | 265.9 | 213.2 | 134.8 | 83.8 | 92.5 | 0.105 |
| 8 | 313.6 | 312.0 | 368.0 | 312.1 | 239.8 | 155.7 | 98.7 | 206.5 | 0.004** |
| 10 | 342.1 | 365.6 | 362.0 | 318.5 | 230.1 | 196.0 | 175.3 | 246.9 | ＜0.001*** |
| 12 | 416.0 | 385.3 | 604.7 | 569.9 | 223.8 | 176.1 | 149.9 | 203.7 | 0.002* |
| 14 | 573.2 | 594.1 | 583.2 | 678.9 | 220.5 | 242.6 | 190.1 | 296.5 | ＜0.001*** |
|  | **MnO_2_-BSA group** | | | | **^211^At-ATE-MnO_2_-BSA** | | | |  |
| 0 | 64.0 | 29.7 | 53.4 | 68.8 | 96.3 | 65.6 | 47.7 | 63.0 | 0.330 |
| 2 | 62.1 | 42.8 | 74.0 | 75.6 | 130.1 | 91.0 | 58.2 | 72.0 | 0.212 |
| 4 | 163.2 | 98.8 | 141.3 | 52.9 | 209.5 | 130.0 | 79.5 | 106.9 | 0.655 |
| 6 | 140.5 | 104.4 | 171.0 | 172.8 | 213.2 | 134.8 | 83.8 | 92.5 | 0.649 |
| 8 | 334.6 | 213.2 | 257.3 | 220.5 | 239.8 | 155.7 | 98.7 | 206.5 | 0.098 |
| 10 | 376.5 | 257.3 | 382.9 | 298.4 | 230.1 | 196.0 | 175.3 | 246.9 | 0.015* |
| 12 | 427.1 | 413.5 | 449.2 | 454.1 | 223.8 | 176.1 | 149.9 | 203.7 | ＜0.001*** |
| 14 | 514.0 | 421.6 | 542.7 | 518.9 | 220.5 | 242.6 | 190.1 | 296.5 | ＜0.001*** |
|  | **^211^At** | | | | **^211^At-ATE-MnO_2_-BSA** | | | |  |
| 0 | 31.5 | 74.4 | 58.7 | 26.1 | 96.3 | 65.6 | 47.7 | 63.0 | 0.229 |
| 2 | 46.9 | 80.8 | 62.9 | 45.6 | 130.1 | 91.0 | 58.2 | 72.0 | 0.154 |
| 4 | 87.5 | 156.8 | 141.7 | 121.8 | 209.5 | 130.0 | 79.5 | 106.9 | 0.891 |
| 6 | 97.5 | 147.8 | 127.5 | 171.5 | 213.2 | 134.8 | 83.8 | 92.5 | 0.885 |
| 8 | 167.4 | 165.6 | 151.7 | 116.8 | 239.8 | 155.7 | 98.7 | 206.5 | 0.481 |
| 10 | 249.7 | 257.3 | 235.4 | 213.2 | 230.1 | 196.0 | 175.3 | 246.9 | 0.205 |
| 12 | 324.5 | 336.5 | 293.6 | 238.2 | 223.8 | 176.1 | 149.9 | 203.7 | 0.007** |
| 14 | 406.6 | 396.1 | 321.4 | 259.2 | 220.5 | 242.6 | 190.1 | 296.5 | 0.039* |
